# Application of a multimodal model integrating optical coherence tomography angiography (OCTA) and radiomics for the identification of high-risk Nonarteritic anterior ischaemic optic neuropathy (NAION) populations and for distinguishing its acute phase

**DOI:** 10.1186/s12886-026-05186-w

**Published:** 2026-08-03

**Authors:** Peixian Yang, Songlin Cui, Yuxin Zhang, Ruijuan Zhao, Xiaoting Xi, Xin Xiong, Luda Chen, Jianfeng He, Jia Ma

**Affiliations:** 1 First Affiliated Hospital of Kunming Medical University, Department of Ophthalmology, Kunming, China; 2 Present Address: Department of Ophthalmology, Affiliated Hospital of North Sichuan Medical College, Nanchong, China; 3 School of Information Engineering and Automation, Kunming University of Science and Technology, Kunming, China

**Keywords:** Non-arteritic anterior ischemic optic neuropathy (Naion), Optical coherence tomography angiography (Octa), Vessel density, Radiomics, Deep learning

## Abstract

**Background:**

Nonarteritic anterior ischaemic optic neuropathy (NAION) is an acute optic neuropathy characterized primarily by sudden vision loss accompanied by visual field defects. Early screening and diagnosis are especially critical because of the diagnostic challenges associated with the initial stages of NAION and its irreversible nature. This study proposes a multimodal diagnostic approach that integrates optical coherence tomography angiography (OCTA) and radiomics for the identification of high-risk NAION populations and for distinguishing its acute phase.

**Methods:**

Without requiring region-of-interest (ROI) delineation, we globally extracted radiomics features from OCTA images and combined them with clinical information. Multilayer perceptron (MLP) models were subsequently developed for three distinct scenarios: single-modality single-region, multimodality single-region, and multimodality multiregion analyses.

**Results:**

The results demonstrate that the multimodality multiregion model, incorporating multiregional OCTA features and clinical information, achieved optimal performance in NAION prediction, with an AUC of 0.996 (95% CI, 0.983–1.000) and an ACC of 0.970. This model also exhibited outstanding performance in differentiating acute-phase NAION from acute-phase optic neuritis (ON), with an AUC of 0.989 (95% CI, 0.966–1.000) and an ACC of 0.944.

**Conclusions:**

By integrating multisource information, the model not only enhances diagnostic accuracy but also provides clinicians with comprehensive decision support perspectives. This advancement facilitates early intervention and personalized treatment strategies, thereby mitigating disease progression and improving prognostic outcomes for NAION patients. This study establishes a novel diagnostic pathway and offers innovative methodologies for the early diagnosis and management of NAION.

## Introduction

Nonarteritic anterior ischaemic optic neuropathy (NAION) is an acute, painless visual impairment characterized clinically by sudden, moderate vision loss in one eye, optic disc oedema, and visual field defects [1]. Epidemiological studies estimate that the annual incidence rate of NAION is 2 to 10 cases per million population. With increasing population ageing, this incidence has demonstrated a progressive upward trend [2]. In NAION patients, ischaemia typically manifests segmentally within the optic disc. This results in sectoral disc oedema during the acute phase, progressing to sectoral optic atrophy characterized by pallor in chronic stages. Since retinal ganglion cell damage postischemia is irreversible, patients consistently develop permanent, dense altitudinal visual field defects. These deficits severely compromise both visual function and quality of life. Importantly, NAION is frequently underdiagnosed or misdiagnosed in early stages. Given that disease progression is irreversible once it is initiated, early screening becomes paramount [3]. The current management of NAION faces two challenges: an incomplete mechanistic understanding of its pathogenesis and a critical lack of effective therapies. Notably, optic neuritis (ON) presenting with acute disc oedema and NAION exhibit substantial overlap in clinical features during the acute phase. This phenotypic similarity contributes to remarkably high misdiagnosis rates, particularly in resource-limited settings [4]. Consequently, affected patients may undergo multiple invasive procedures, including excessive MRI scanning, unwarranted lumbar punctures, and nonindicated administration of intravenous corticosteroids. This cascade of low-yield interventions compounds healthcare costs while exposing patients to preventable iatrogenic risks [5].

With the advancement of artificial intelligence (AI), its applications in medical diagnostics have rapidly emerged, particularly in radiomics-driven computer-aided diagnosis (CAD) systems that extract quantifiable biomarkers from medical images. Bunod et al. [6] provided significant inspiration: integrating more information during diagnostic model development leads to higher diagnostic accuracy and greater model robustness. Therefore, for the OCTA imaging data, we collected not only radial peripapillary capillaries (RPC) and superficial regions but also the choroid. Moreover, we incorporated clinical information related to NAION onset for auxiliary diagnosis. The contributions of this study are as follows: (i) Utilizing contralateral unaffected eyes of NAION patients to identify high-risk NAION populations, screen high-risk populations, and differentiate acute NAION from acute disc oedema in ON patients using OCTA; (ii) employing radiomics to extract global features without delineating regions of interest (ROIs) on individual OCTA layers; and (iii) establishing diagnostic models using radiomics features from individual OCTA layers and constructing multimodal multisite models combining features with clinical information. The multimodal approach achieved precise screening of high-risk NAION populations in the general population and accurate acute-phase differential diagnosis for patients with disc oedema, outperforming traditional single-modality methods and surpassing clinical expert performance.

This research provides a potentially interpretable, highly robust AI-assisted diagnostic paradigm for early NAION screening and rapid differentiation of complex optic neuropathies. The radiomics features used in our model can be linked to clinically meaningful imaging biomarkers, offering greater transparency than fully black-box deep learning models do, although we acknowledge that the model is still a data-driven classifier that cannot fully replicate the clinical reasoning process of human experts.

## Methods

With technological advancements, AI has emerged. In neuro-ophthalmic clinical practice, AI technologies have established a multitiered technical framework, with core contributions manifested in three domains: early lesion screening, diagnostic efficiency optimization, and clinical resource allocation optimization. Machine learning-based analysis of fundus imaging and optical coherence tomography (OCT) data enables effective discernment of subtle pathological signs such as ON and retinal nerve fibre layer (RNFL) thinning [7]. Establishing a dynamic assessment system for optic neuropathies enhances the ability to predict disease progression. In primary care settings, compared with conventional manual approaches, AI-assisted platforms significantly reduce physicians’ image interpretation time while improving diagnostic accuracy rates. Moreover, mobile-based intelligent screening systems play a vital role in telemedicine [8]. OCTA, a noninvasive novel fundus imaging technique, enables high-resolution visualization of blood flow across retinal layers. Our collected patient OCTA datasets consistently encompass three key vascular compartments: the choroidal layer, RPC layer, and superficial retinal layer. NAION and ON exhibit distinct pathological signatures within specific OCTA compartments [9–12], providing localized anatomical perspectives. This necessitates effective feature extraction from OCTA data for subsequent analytical tasks, culminating in the development of high-accuracy, robust diagnostic models integrated with clinical information.

We initially conducted preliminary clinical validations, which demonstrated that discernible differences exist between acute-phase NAION and acute-phase ON, and between the contralateral healthy eyes of NAION patients and the eyes of normal individuals, all of which can be identified via OCTA.

### Descriptive baseline OCTA findings

#### Demographics and baseline characteristics

This cross-sectional study enrolled 106 patients with unilateral NAION (106 eyes). Affected eyes were categorized into the following groups according to disease duration: Group A (acute phase): 37 eyes (duration ≤ 2 weeks; male/female = 21/16; age 51.37 ± 12.13 years) and Group C (chronic phase): 69 eyes (duration > 6 months; male/female = 39/30; age 52.06 ± 13.05 years). The contralateral healthy eyes of the patients (106 eyes) constituted Group H. Additionally, 25 age- and sex-matched healthy volunteers (50 eyes) without diabetes, hypertension, dyslipidaemia, or prior vitreoretinal surgery were recruited as Group N (male/female = 11/14; age 50.28 ± 10.21 years). For reference, 31 eyes with acute papilledema due to ON were included (male/female = 12/19; age 49.55 ± 13.76 years). Statistical comparisons: There were no significant intergroup differences in age (ANOVA, *P* = 0.621) or sex distribution (χ² test, *P* = 0.473) Table 1.

**Table 1:** Analysis of demographics and baseline characteristics

	Age	Sex (male/female)
NAION Acute	51.37 ± 12.13	21/16
NAION Chronic	52.06 ± 13.05	39/30
ON Acute	49.55 ± 13.76	12/19
Normal	50.28 ± 10.21	11/14
*P*_*age*_ (ANOVA)	0.621	\
*P*_sex_ (χ²)	\	0.473

Table 1 All the data are presented as the mean ± SD

All the NAION patients included in this study met the following criteria (Wei SH. Expert Consensus on the Diagnosis and Treatment of Non-Arteritic Anterior Ischaemic Optic Neuropathy in China (2015). Chin J Ophthalmol. 2015;000(5):4 [13]):

1. Sudden painless visual acuity loss and/or visual field defects;
2. Fundus examination reveals segmental optic disc oedema in the acute phase, and the oedema resolves with optic disc pallor in the atrophic phase;
3. Typically presents with arcuate or hemifield visual field defects connected to the physiological blind spot, most commonly affecting the inferior visual field;
4. Fundus fluorescein angiography shows delayed perfusion;
5. Age ≥ 45 years.

All ON patients included in this study met the criteria outlined by Petzold A et al. (Diagnosis and classification of ON. Lancet Neurol. 2022;21(12):1120–1134 [14]). as follows:

1. Subacute visual decline, ocular pain, colour vision and contrast sensitivity disorders, and relative afferent pupillary defects (RAPDs);
2. Fundus examination revealed optic disc oedema;
3. Enhanced optic nerve MRI showed abnormalities in the optic nerve of the affected eye during the acute phase;
4. Age ≥ 45 years.
5. All ON patients were idiopathic.

The exclusion criteria were as follows:

1. Patients who are currently suffering from or have a history of diseases such as intraorbital or intracranial space-occupying lesions that affect the diagnosis of NAION;
2. Patients with diseases causing optic nerve or retinal damage or a history of ocular laser treatment or intraocular surgery;
3. Patients with intraocular pressure monitoring results > 21 mmHg within 24 h, a cup-to-disc ratio > 0.4, or a history of glaucoma;
4. Patients with opacity of the refractive media of the affected eye that affects image clarity;
5. Patients who cannot persist in completing examinations or have low compliance, leading to unreliable examination results.
6. In the normal control group, individuals with diabetes, hypertension, hyperlipidaemia, or a history of fundus surgery that affects optic disc blood flow density.

#### Conventional statistical analysis of OCTA parameters

##### Optic disc characteristics on OCTA in eyes with acute NAION versus acute ON

**Fig. 1:**
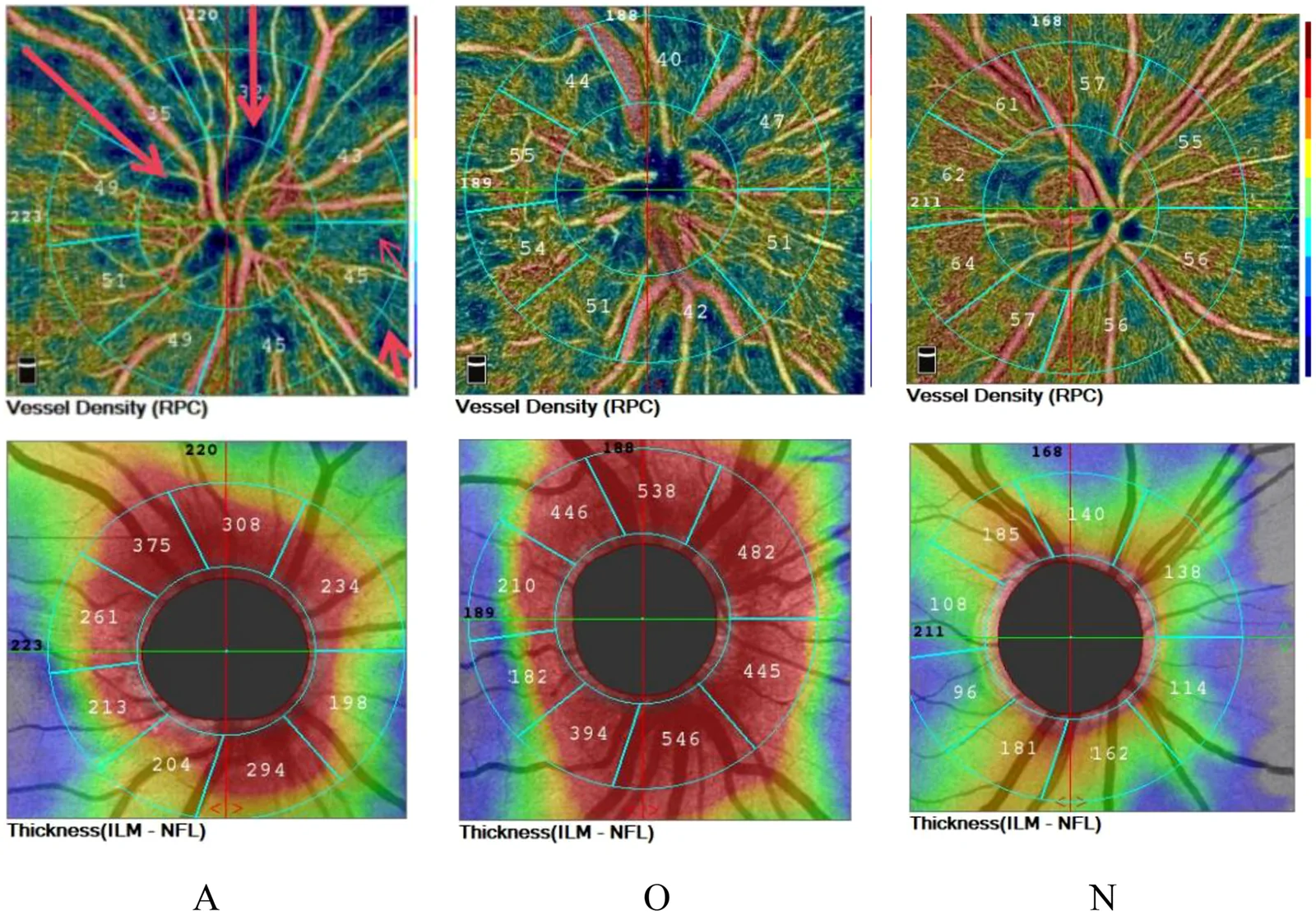
Representative RPC vessel density and pRNFL thickness in a study subject. Group A: acute NAION; Group O: acute ON; Group N: normal. Top panel: optic disc vessel density (VD); Bottom panel: peripapillary retinal nerve fibre layer (pRNFL) thickness

Eyes with acute NAION (Group A) demonstrated a significant reduction in vessel density in the regions indicated by arrows compared with those with acute ON (Group O) (Fig. 1).

As shown in Table 2, compared with eyes with acute ON (Group O), those with acute NAION (Group A) exhibited reduced vessel density (VD) in the following regions: the whole optic disc (VD_W_), inside disc (VD_D_), superior hemisphere (VD_S_), and inferior hemisphere (VD_I_). Significant intergroup differences were observed in the VD_W_ and VD_S_ (*P* < 0.05), whereas the VD_D_ and VD_I_ were not significantly different (*P* > 0.05). The peripapillary RNFL (pRNFL) thickness was significantly greater in Group A than in Group O (*P* < 0.05).

**Table 2:** Parameters and group comparisons among the NAION, contralateral, normal, and optic neuritis eyes

	A	C	*N*	H	O	A-C	A-*N*	A-H	C-*N*	C-H	A-O	*N*-H
	average					*P*_1_	*P*_2_	*P*_3_	*P*_4_	*P*_5_	*P*_6_	*P*_7_
VD_W_ (%)	41.89 ± 3.34	36.60 ± 6.18	46.12 ± 4.00	44.23 ± 4.55	44.73 ± 5.04	***P	0.003	0.045	***P	***P	0.015*	0.024
VD_D_ (%)	42.03 ± 4.07	40.45 ± 5.53	43.22 ± 7.60	42.02 ± 6.99	44.29 ± 7.47	0.383	1.000	0.420	0.048	***P	0.159	0.394
VD_S_ (%)	42.14 ± 5.52	35.57 ± 8.75	50.37 ± 5.24	47.24 ± 6.75	46.76 ± 7.35	***P	***P	0.002	***P	***P	0.010*	0.008
VD_I_ (%)	45.86 ± 4.12	37.82 ± 8.26	49.33 ± 5.19	48.11 ± 4.27	46.91 ± 6.20	***P	0.077	0.090	***P	***P	0.454	0.210
pRNFL (µm)	247.00 ± 60.04	93.10 ± 30.25	111.50 ± 19.23	121.54 ± 21.90	196.81 ± 97.54	***P	***P	***P	0.801	***P	0.012	0.023
Superior pRNFL (µm)	242.93 ± 64.27	79.68 ± 32.94	111.42 ± 19.49	120.72 ± 24.81	192.92 ± 98.45	***P	***P	***P	0.851	***P	0.009	0.042
Inferior pRNFL (µm)	255.81 ± 70.97	108.36 ± 35.91	109.98 ± 20.23	123.00 ± 20.60	204.34 ± 104.68	***P	***P	***P	0.870	0.002	0.004	0.049

Note: All the data are presented as the mean ± SD. Statistical significance levels: *P < 0.05, **P < 0.01, *P < 0.001. A t test was used. Intergroup comparisons: P_1_ (A vs. C), P_2_ (A vs. N), P_3_ (A vs. H), P_4_ (C vs. N), P_5_ (C vs. H), P_6_ (A vs. O), P_7_ (N vs. H). VD: vessel density; VD_W_: whole-image vessel density; VD_D_: inside-disc vessel density; VD_S_: superior-hemi vessel density; VD_I_: inferior-hemi vessel density. pRNFL: peripapillary retinal nerve fibre layer

When acute NAION eyes (Group A) were compared with normal control eyes (Group N), Group A demonstrated the following:

(i) Decreased VD in all parameters (VD_W_, VD_D_, VD_S_, VD_I_).
(ii) Increased pRNFL thickness (average, superior, and inferior).

Significant differences (*P* < 0.05) were found in the VD_W_, VD_S_, VD_I_, superior pRNFL, inferior pRNFL, and average pRNFL, but not in the VD_D_ (*P* > 0.05).

Point-biserial correlation analysis (Table 3) revealed the following:

(i) A significant positive correlation between vessel density and disease category (coded as NAION = 1, ON = 0), indicating that higher vessel density is associated with ON;
(ii) A significant negative correlation between pRNFL thickness and disease category, suggesting that increased pRNFL thickness predicts NAION.

**Fig. 2:**
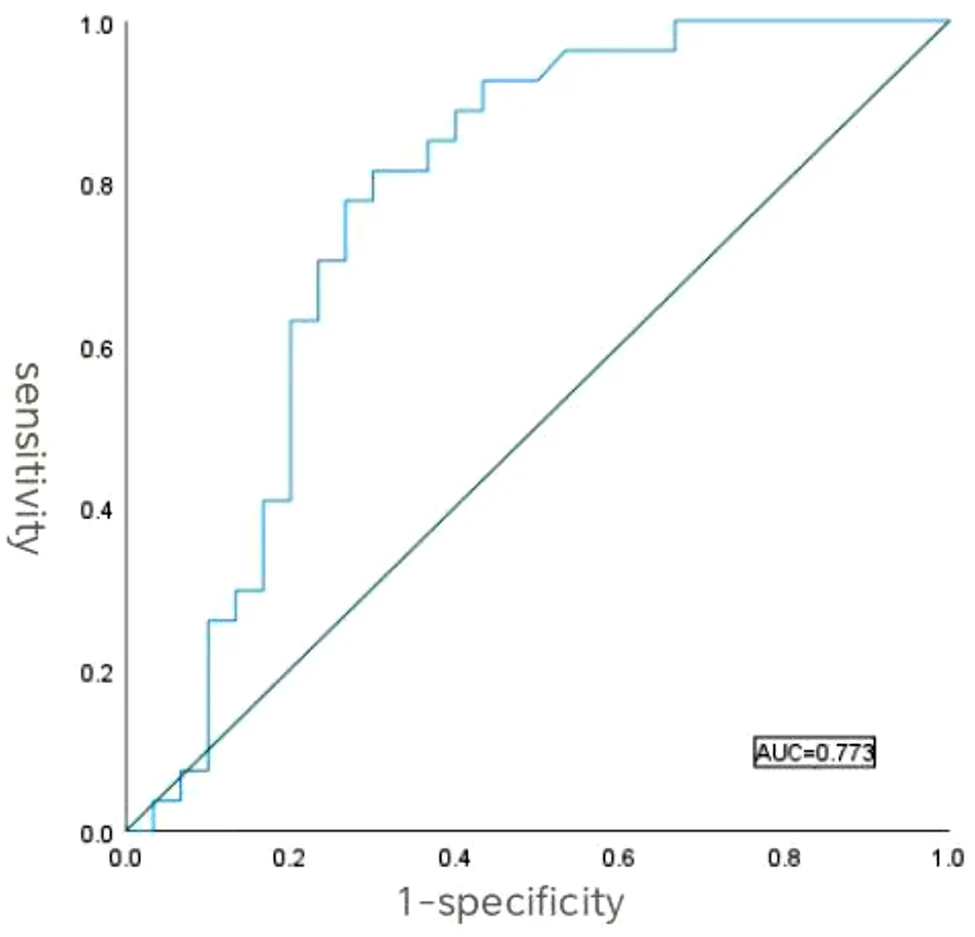
ROC analysis of OCTA parameters for acute NAION and ON discrimination. ROC curve analysis of conventional OCTA parameters for differentiating NAION from acute ON

Binary logistic regression-derived predictive values were used to construct an ROC curve (Fig. 2), yielding an AUC of 0.773 (95% CI: 0.691–0.855). This model facilitates differentiation between acute NAION and ON in clinical practice.

**Table 3:** Point–biserial correlation analysis of vascular parameters between acute NAION and acute ON

	VD_W_ (%)	VD_S_ (%)	VD_I_ (%)	Aeverage pRNFL (µm)	Superior pRNFL (µm)	Inferior pRNFL (µm)
*P*	0.020	0.012	0.438	0.010	0.011	0.019
*R*	0.300	0.332	0.105	-0.341	-0.335	-0.309

##### OCTA optic disc characteristics in NAION fellow eyes versus normal eyes

**Fig. 3:**
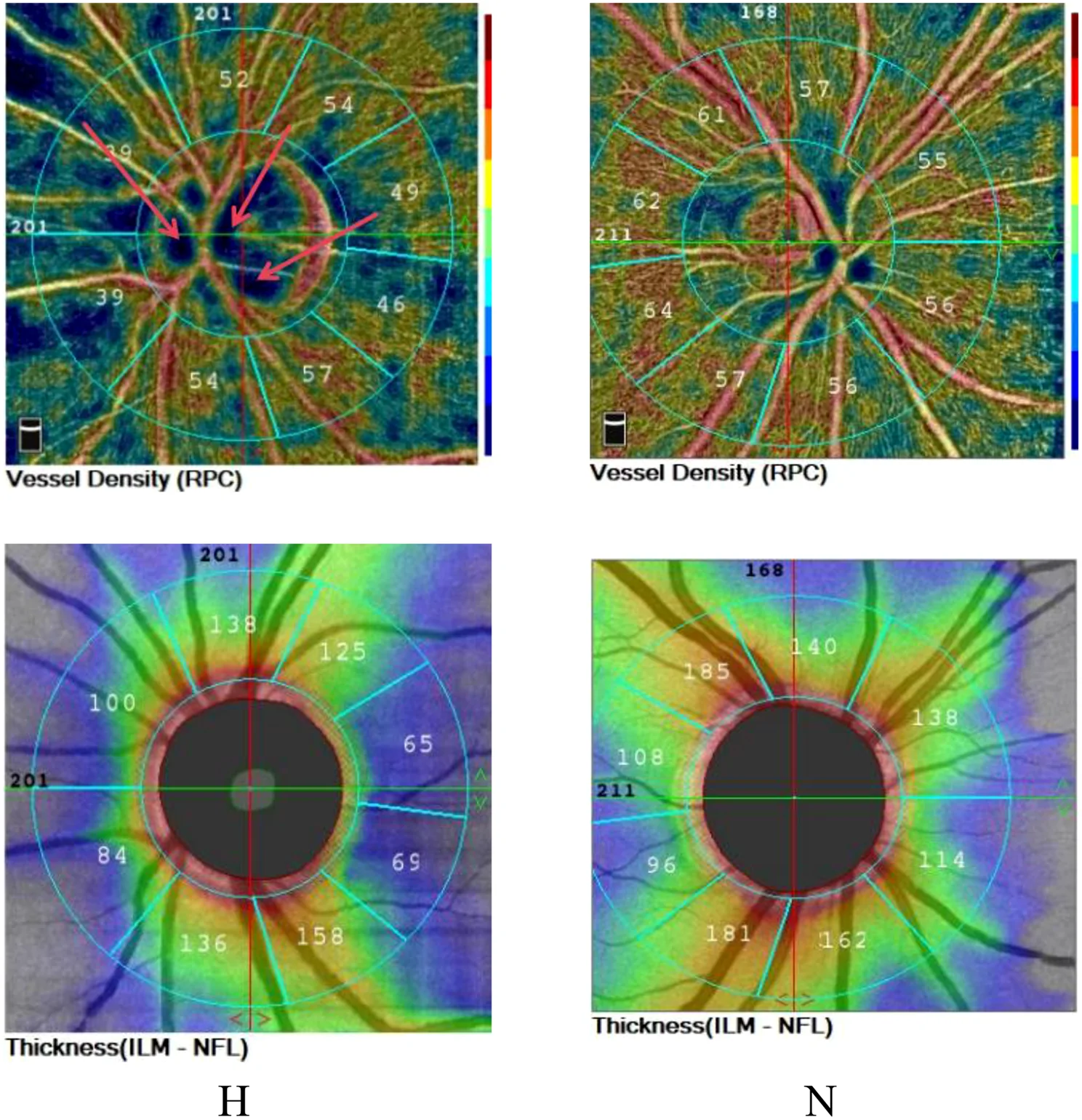
RPC Disc Vessel Density and pRNFL Thickness in the Study Subjects. H: Contralateral healthy eyes of unilateral NAION patients; N: Normal control eyes. Top: Disc vessel density; Bottom: pRNFL thickness

Compared with normal eyes (Group N), fellow eyes with NAION (Group H) presented significantly reduced vessel density in the arrow-indicated regions (Fig. 3).

The results in Table 2 demonstrate that when NAION fellow eyes (Group H) were compared with normal eyes (Group N), the vessel density decreased in all the sectors (VD_W_, VD_D_, VD_S_, and VD_I_), with significant decreases in the VD_W_ and VD_S_ (*P* < 0.05) and a significant increase in the pRNFL thickness (global, superior, inferior; all *P* < 0.05). Point–biserial correlation analysis revealed significant positive correlations between disease category (fellow eyes = 1, normal eyes = 0) and the VD_W_, global pRNFL, superior pRNFL, and inferior pRNFL (higher values associated with normal eyes).

A nonsignificant negative correlation was observed for all VD_S_*(P* > 0.05).

**Fig. 4:**
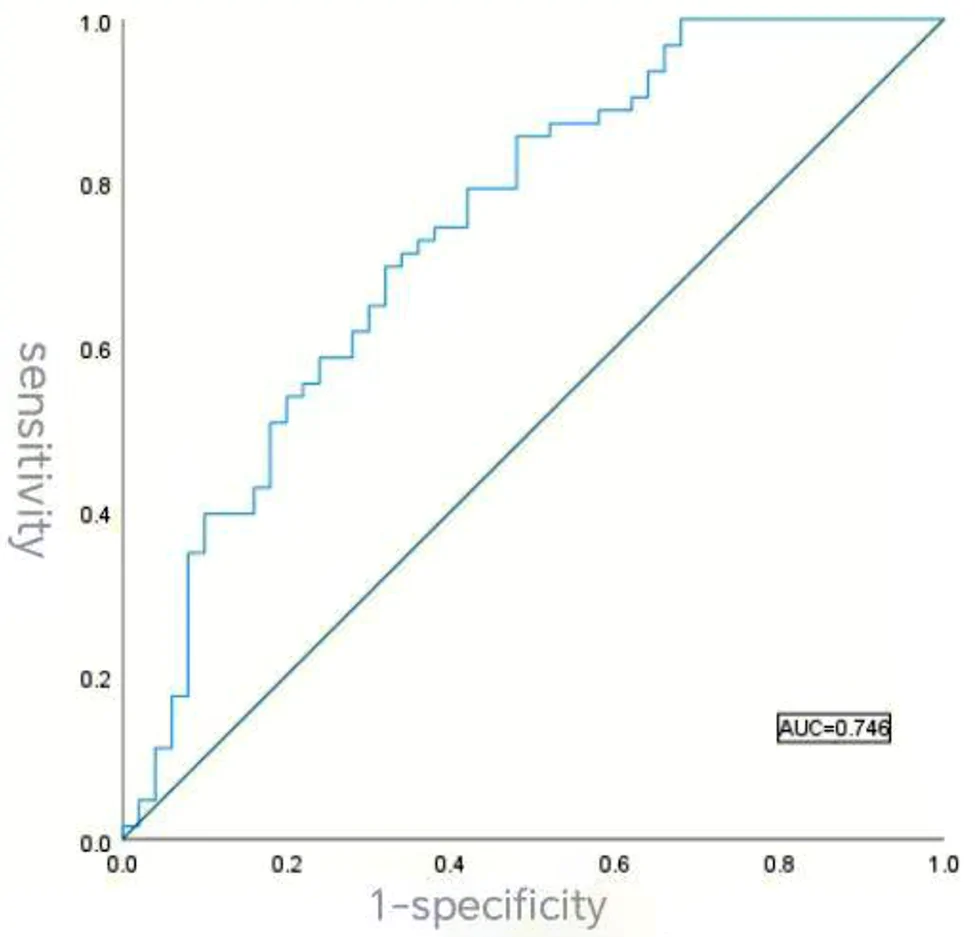
Discriminative Capacity of Optic Disc Characteristics between NAION Fellow Eyes and Normal Controls

Binary logistic regression was conducted using the VD_W_, superior pRNFL, inferior pRNFL, and average pRNFL generated predictive values. The resulting (Table 4) ROC curve (Fig. 4) had an AUC of 0.746 (95% CI: 0.654–0.838). This model serves as a valuable screening tool for identifying high-risk NAION populations.

**Table 4:** Correlation analysis for discriminating H vs. N eyes: VD and pRNFL parameters

	VD_W_ (%)	VD_S_ (%)	pRNFL (µm)	Superior pRNFL (µm)	Inferior pRNFL (µm)
*P*	0.041	0.954	0.001	0.001	0.001
*R*	0.136	-0.05	0.300	0.331	0.345

##### Optic disc features in chronic NAION versus acute NAION, contralateral unaffected eyes, and normal control eyes

**Fig. 5:**
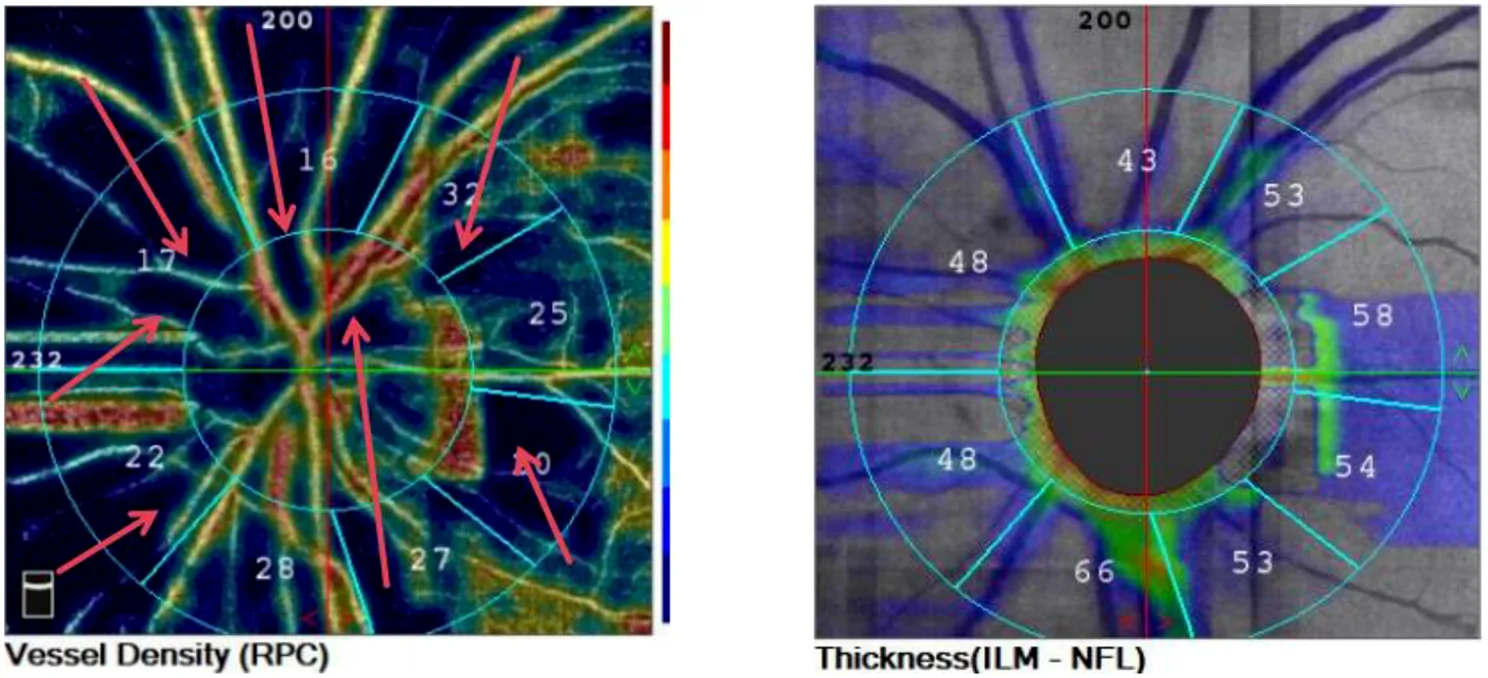
Representative vascular and structural features of the optic disc in chronic NAION

In chronic NAION eyes (Group C), a distinct area of vascular density loss was clearly observed in the region indicated by the red arrow (Fig. 5). As shown in Table 2, compared with acute NAION eyes, Group C exhibited significantly reduced blood flow in terms of the VD_W_, VD_D_, VD_S_, and VD_I_ (Group A), with the reductions in the VD_W_, VD_S_, and VD_I_ reaching statistical significance (*P* < 0.05). The pRNFL thickness, including both the superior and inferior quadrant thicknesses, was significantly lower in Group C than in Group A (*P* < 0.05). Compared with the contralateral unaffected eyes (Group H), Group C demonstrated significantly reduced blood flow in all measured parameters (VD_W_, VD_D_, VD_S_, and VD_I_; *P* < 0.05). Both overall pRNFL thickness and superior pRNFL thickness were significantly lower in Group C than in Group H (*P* < 0.05), although the reduction in inferior pRNFL thickness did not reach statisctical significance. Relative to normal control eyes (Group N), Group C showed slightly but significantly lower blood flow values in terms of the VD_W_, VD_D_, VD_S_, and VD_I_ (*P* < 0.05). While overall pRNFL thickness and superior/inferior pRNFL thickness were numerically lower in Group C than in Group N, these differences were not statistically significant (*P* > 0.05).

### Model development

**Fig. 6:**
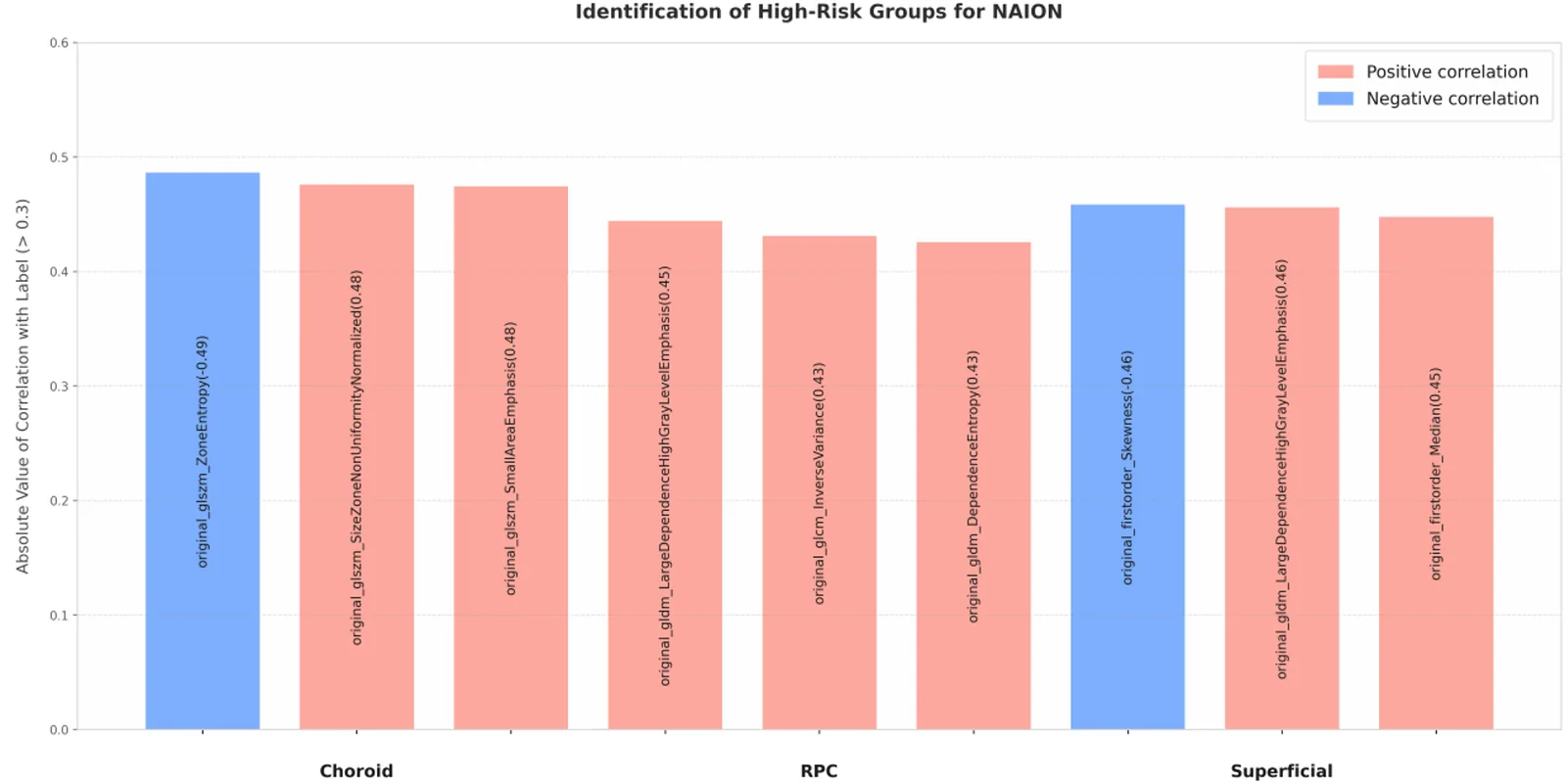
Workflow of OCTA radiomics feature extraction and screening. Global masks are generated for all OCTA scan layers to extract comprehensive 2D radiomics features. Features with absolute correlation coefficients above the threshold of |ρ| > 0.3 are retained through task-specific, layer-wise screening

**Fig. 7:**
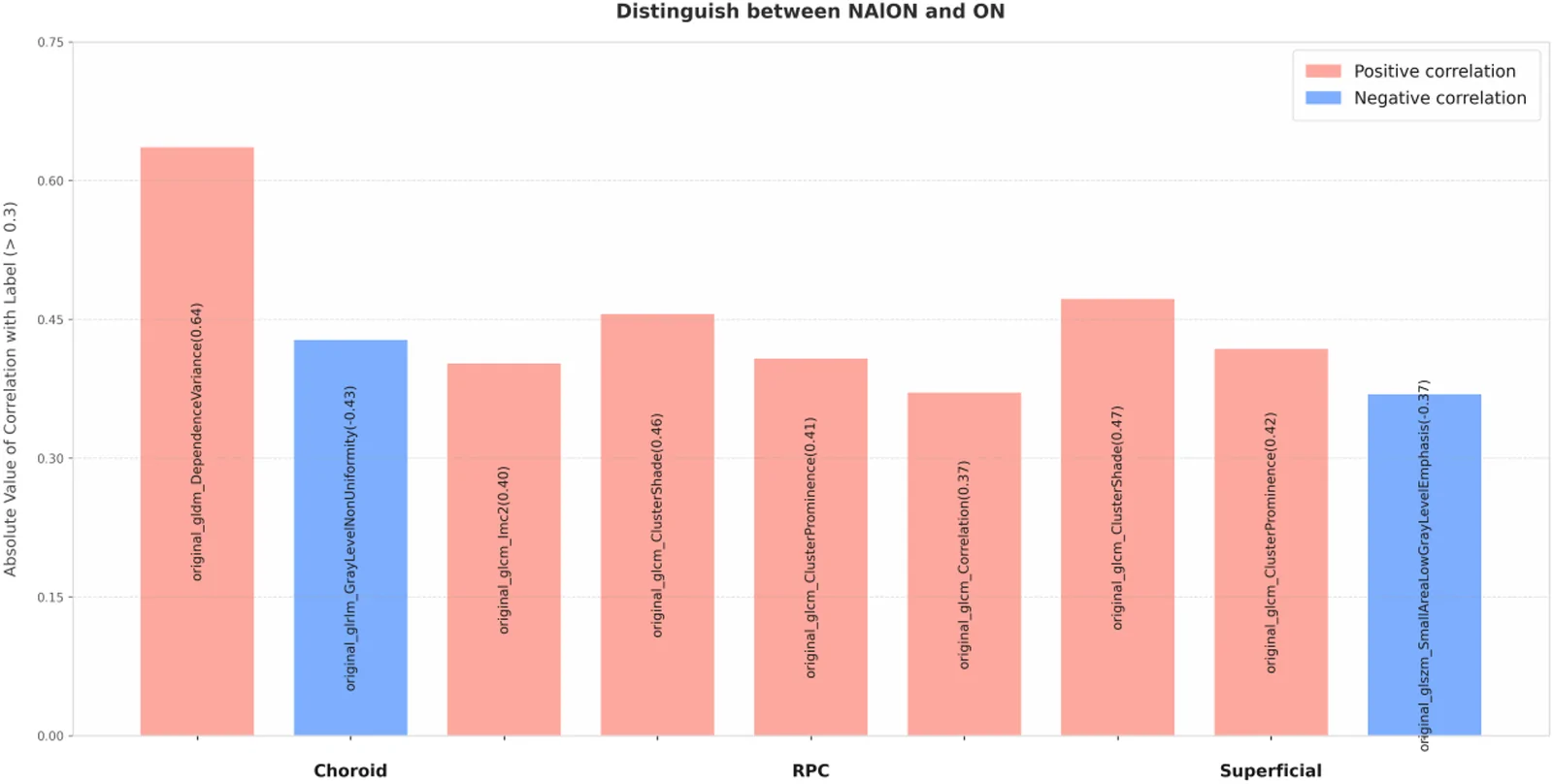
Radiomics feature correlations for NAION and ON differentiation

**Fig. 8:**
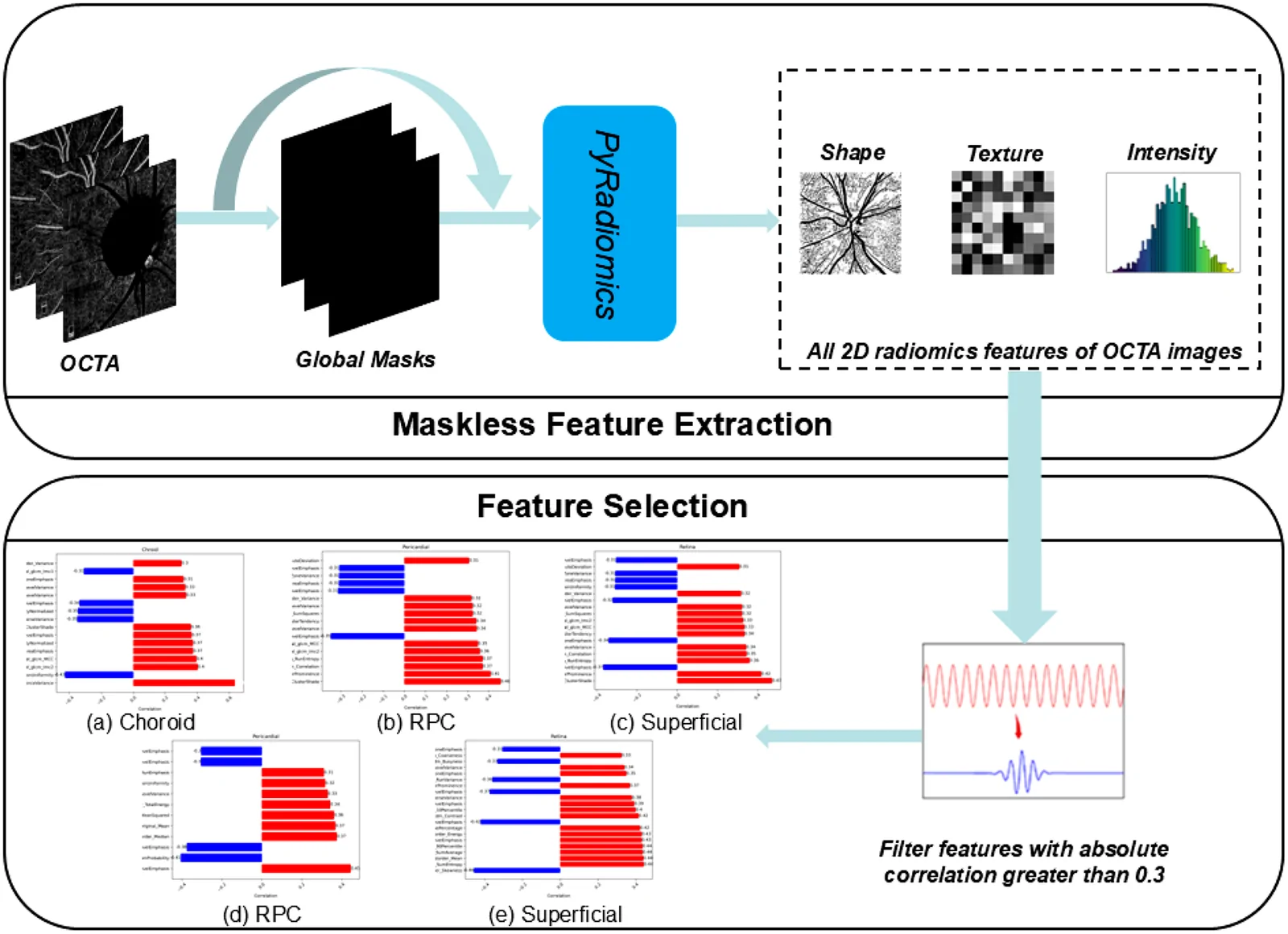
OCTA radiomics features for the discrimination between acute-phase NAION and acute-phase ON Eyes

**Fig. 9:**
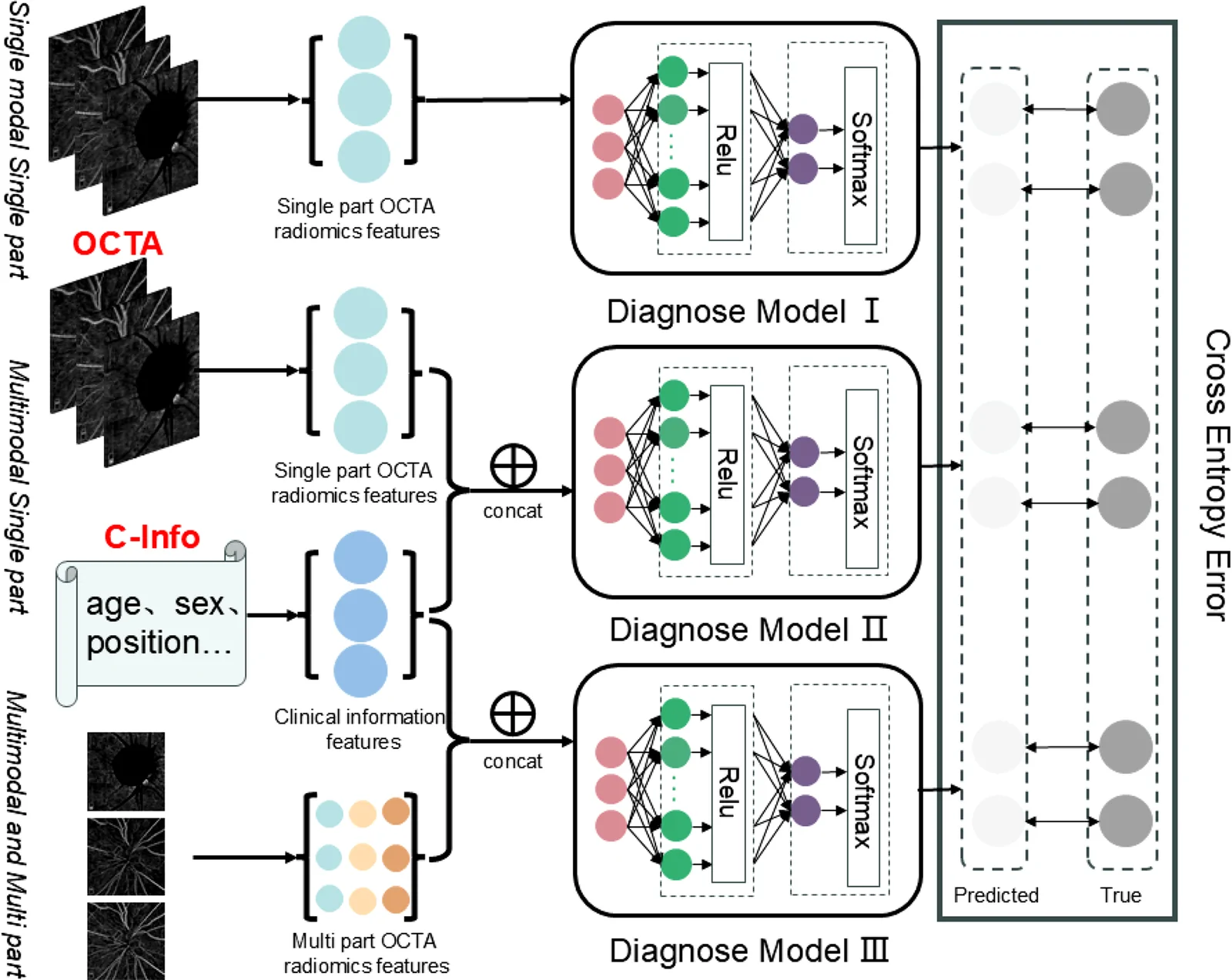
OCTA radiomics features for the identification groups at high risk for NAION

Given that vessels on OCTA are represented by grayscale values distinct from those of surrounding tissues and that NAION-related pathological states manifest physiologically as microvascular density alterations, radiomics enables the derivation of 2D image features through pixel-value computations (Fig. 6). However, radiomic feature extraction requires region-of-interest (ROI) delineation. This proves challenging in peripapillary capillary and superficial retinal OCTA layers because of the extensive distribution and low feature saliency of both the macrovasculature and microvasculature. Furthermore, ROI delineation demands specialized clinicians, constituting a labour-intensive process. To address this, we generate uniformly sized global masks for all OCTA layers prior to extracting comprehensive radiomics features, with the workflow illustrated in Fig. 7. Not all computed radiomics features are relevant for the identification of high-risk NAION populations or differentiation between acute-phase NAION and acute-phase ON. Redundant features with low correlations may compromise model performance—a critical advantage over deep learning approaches given the scarcity of clinical NAION data—as it permits manual curation of clinically interpretable features with strong biological relevance (Figs. 8 and 9). We subsequently conducted correlation-based feature screening on all the radiomics features.

Multilayer perceptron (MLP) models were developed for three distinct scenarios: single-modality single-region, multimodality single-region, and multimodality multiregion analyses. To ensure transparency and avoid information leakage, all preprocessing and feature selection steps were performed strictly within each cross-validation fold. For the training set of each fold, we first used mutual information to select features significantly associated with the outcome label. Then, features with absolute correlation coefficients exceeding the threshold of |ρ| > 0.3 were selected through task-specific and layerwise screening to reduce feature dimensionality. To address potential class imbalance in the training data, we applied class weights inversely proportional to class frequencies in the loss function, ensuring that the model did not bias towards the majority class. This fold-specific processing ensured that no information from the test fold was used in any step of model training or feature selection, fully preventing data leakage.

The feature-selection process was performed strictly within each cross-validation fold to prevent data leakage, following a two-stage approach:

Stage 1: Mutual information screening: For the training set of each fold, we first calculated the mutual information between each radiomic feature and the outcome label. Features with mutual information values below a predefined threshold were excluded to remove noninformative features.

Stage 2: Correlation-based filtering: Among the remaining features, we computed pairwise Pearson correlation coefficients. Features with absolute correlation coefficients |ρ| > 0.3 with the outcome label were retained for model training. This threshold was selected to balance feature dimensionality reduction with information preservation, ensuring that only clinically and statistically relevant features entered the model.

This two-stage feature selection was performed independently for each OCTA layer (choroid, RPC, and superficial) and for each classification task, resulting in task-specific and layer-specific feature sets.

To address potential class imbalance in the training data, we applied class weights in the cross-entropy loss function. The class weight for each class was set to be inversely proportional to its frequency in the training set of each fold. This weighting strategy ensures that the model does not bias towards the majority class and that misclassification of the minority class has a proportionally higher penalty.

We implemented a modified 5-fold cross-validation protocol with enhanced stringency. The training-to-test split ratio was set at 7:3 (rather than the conventional 8:2) to rigorously evaluate the generalization capability and confirm the absence of overfitting.

Critically, all cross-validation splits were performed at the patient level rather than at the eye level. For patients with both eyes included in the dataset (e.g., the affected eye and the contralateral eye of unilateral NAION patients), all the eyes from the same patient were assigned to the same cross-validation fold. This patient-level stratification ensures that the training and test sets are fully independent at the subject level, eliminating information leakage that could arise from within-subject correlations between paired eyes.

All preprocessing steps, including feature standardization (Z score normalization) and feature selection, were performed exclusively on the training set of each fold, and the resulting transformation parameters were then applied to the corresponding test set. This ensures that no information from the test fold was used in any step of model training or feature engineering.

Clinically, physicians integrate multimodal examination data with clinical information for diagnosis. Similarly, to train an accurate and robust diagnostic model, we collected NAION-relevant clinical parameters from patients, as summarized in Table 5 General Information of NAION Patients. Following radiomic feature extraction across OCTA scan layers, feature standardization was performed to ensure uniform feature scaling, preventing model dominance by features with wider numerical ranges. The standardization procedure and the computational workflow and results are detailed below:

1-1 $$\eqalign{ & {{\rm{f}}_{{\rm{Chroid}}}}{\rm{,}}{{\rm{f}}_{{\rm{RPC}}}}{\rm{,}}{{\rm{f}}_{{\rm{Superficial}}}}{\rm{ = STD(Radiomics}} \cr & {\rm{(OCT}}{{\rm{A}}_{{\rm{Chroid}}}}{\rm{,OCT}}{{\rm{A}}_{{\rm{RPC}}}}{\rm{,OCT}}{{\rm{A}}_{{\rm{Superficial}}}}{\rm{))}} \cr}$$

**Table 5:** General information of the NAION patients

	Healthy NAION (*n* = 180)	Normal (*n* = 147)	Acute NAION (*n* = 87)	Acute ON (*n* = 93)	Healthy NAION vs. Normal (*P*)	Acute NAION vs. Acute ON (*P*)
Age	50.09 ± 11.43	50.28 ± 10.21	51.37 ± 12.13	49.55 ± 13.76	0.507	0.428
Sex male/female	132/48	66/81	60/27	36/57	0.820	< 0.001**
Diabetes	63(35.1%)	52(35.2%)	30(34.9%)	14(15.7%)	0.975	< 0.001*
Hypertension	67(37.5%)	66(45.1%)	32(36.8%)	10(10.5%)	0.210	0.008*
Hyperlipidaemia	82(45.6%)	50(33.7%)	38(44.3%)	25(26.0%)	0.580	0.023
Snore	14480.2%)	55(37.5%)	66(75.5%)	34(36.8%)	0.320	0.015
Smoke	46(25.6%)	32(22.1%)	21(24.5%)	10(10.5%)	0.187	0.012
Drink alcohol	56(31.1%)	48(32.6%)	25(29.2%)	10(10.5%)	0.650	0.110

All numerical data are expressed as the mean ± standard deviation (mean ± SD); *P < 0.01, **P < 0.001

MLPs [15] are feedforward neural networks comprising an input layer, one or more hidden layers, and an output layer. We implemented a modified 5-fold cross-validation protocol with enhanced stringency. The training-to-test split ratio was deliberately set at 7:3 (compared with the conventional 8:2 ratio) to rigorously evaluate the generalization capability and confirm the absence of overfitting. Unlike traditional machine learning algorithms (e.g., random forest, K-means, and support vector machine (SVM)), MLPs possess learnable parameters. The incorporation of ReLU activation functions [16] enables nonlinear feature representations, facilitating the development of customized nonlinear diagnostic models for complex disease classification with high accuracy. Key advantages include computational efficiency and the mitigation of vanishing gradients through constant gradient propagation in the positive domain. The ReLU function is formally defined as follows:

1-2 $$\:\begin{array}{c}\mathrm{R}\mathrm{e}\mathrm{L}\mathrm{U}\mathrm{(}\mathrm{Z}\mathrm{)}\mathrm{=}\mathrm{M}\mathrm{A}\mathrm{X}\mathrm{(}\mathrm{0}\mathrm{,}\mathrm{F}\mathrm{)}\end{array}$$

where F denotes the input features comprising both radiomics features from distinct OCTA scan layers and patients’ clinical parameters. After processing through the MLP’s input and multiple hidden layers, the output layer coupled with a softmax function yields class prediction probabilities. We progressively constructed three integrated models: (i) a single-modality single-layer model, i.e., an MLP-based diagnostic model using radiomics features selected via correlation filtering (|*P*| > 0.3) from individual OCTA layers; (ii) a multimodal single-layer model, i.e., an MLP architecture incorporating both optimally performing OCTA layer features (from model i) and corresponding clinical parameters; and (iii) a multimodal multilayer model, i.e., an MLP that integrates radiomics features from multiple OCTA layers with clinical parameters (as detailed in Fig. 3). In the model, b denotes the bias vector. The mathematical formulations for each model are defined as follows:

1-3 $$\eqalign{ & {{\rm{M}}_{{\rm{dia1}}}}{\rm{ = MLP(}}{{\rm{f}}_{{\rm{Choroid}}}}{\rm{|}}{{\rm{f}}_{{\rm{RPC}}}}{\rm{|}}{{\rm{f}}_{{\rm{Superficial}}}}{\rm{)}} \cr & {\rm{ = Softmax(ReLU(}}{{\rm{W}}_{\rm{1}}}{\rm{(}}{{\rm{f}}_{{\rm{Choroid}}}}{\rm{|}}{{\rm{f}}_{{\rm{RPC}}}}{\rm{|}}{{\rm{f}}_{{\rm{Superficial}}}}{\rm{) + b))}} \cr} $$

1-4 $$\eqalign{ & {{\rm{M}}_{{\rm{dia2}}}}{\rm{ = MLP(}}{{\rm{f}}_{{\rm{Choroid}}}}{\rm{|}}{{\rm{f}}_{{\rm{RPC}}}}{\rm{|}}{{\rm{f}}_{{\rm{Superficial}}}}{\rm{,}}{{\rm{C}}_{{\rm{Info}}}}{\rm{)}} \cr & {\rm{ = Softmax(ReLU(}}{{\rm{W}}_{\rm{1}}}{\rm{(}}{{\rm{f}}_{{\rm{Choroid}}}}{\rm{|}}{{\rm{f}}_{{\rm{RPC}}}}{\rm{|}}{{\rm{f}}_{{\rm{Superficial}}}}{\rm{)}} \cr & {\rm{ + }}{{\rm{W}}_{\rm{2}}}{\rm{(}}{{\rm{C}}_{{\rm{Info}}}}{\rm{) + b)}} \cr}$$

1-5 $$\eqalign{ & {\rm{}}{{\rm{M}}_{{\rm{dia3}}}}{\rm{ = MLP(}}{{\rm{f}}_{{\rm{Choroid}}}}{\rm{,}}{{\rm{f}}_{{\rm{RPC}}}}{\rm{,}}{{\rm{f}}_{{\rm{Superficial}}}}{\rm{,}}{{\rm{C}}_{{\rm{Info}}}}{\rm{)}} \cr & {\rm{ = Softmax(ReLU(}}{{\rm{W}}_{\rm{1}}}{\rm{(}}{{\rm{f}}_{{\rm{Choroid}}}}{\rm{,}}{{\rm{f}}_{{\rm{RPC}}}}{\rm{,}}{{\rm{f}}_{{\rm{Superficial}}}}{\rm{)}} \cr & {\rm{ + }}{{\rm{W}}_{\rm{2}}}{\rm{(}}{{\rm{C}}_{{\rm{Info}}}}{\rm{) + b))}} \cr}$$

Figure 7 illustrates three diagnostic models constructed using MLPs: (i) a single-modality single-layer model employing radiomics features extracted and filtered via correlation analysis (|P| > 0.3) from individual OCTA scan layers; (ii) a multimodal single-layer model integrating optimal-performing OCTA layer features with clinical parameters; and (iii) a multimodal multilayer model combining radiomics features from multiple OCTA layers with patients’ clinical data.

To comprehensively evaluate the diagnostic efficacy of the models across both classification tasks, we employed the following performance metrics for holistic assessment: accuracy (ACC), area under the receiver operating characteristic curve (AUC), recall (REC), specificity (SPE), precision (PREC) and F1 score (F1). These metrics collectively provide a multidimensional perspective on model performance, encompassing all critical aspects of diagnostic accuracy. Each metric facilitates in-depth analysis of distinct dimensions of model effectiveness, ensuring a rigorous evaluation of clinical diagnostic capabilities.

## Data collection

This retrospective study utilized deidentified medical images stripped of all personally identifiable information. The study was conducted in strict accordance with the ethical principles of the Declaration of Helsinki and the protocol was approved by the Institutional Review Board of our hospital (Approval No. (2023) Lunshen-L 29). Support for this project was provided by the Yunnan Province “Xingdian Talent Support Program” (XDYC-YLWS-2023-0039) through its Medical and Health Talent Special Fund. All participants provided written informed consent after comprehensive explanation of the study procedures, ensuring voluntary participation with fully protected rights.

We enrolled patients from the Ophthalmology Department of the First Affiliated Hospital of Kunming Medical University between January 2024 and January 2025 who met the “Expert Consensus on the Diagnosis and Treatment of Nonarteritic Anterior Ischaemic Optic Neuropathy in China (2015)” criteria. All ON patients included in this study met the diagnosis and classification criteria for ON.

A diagnosis of acute/chronic NAION or ON with disc oedema was confirmed by neuro-ophthalmologists. Age- and sex-matched healthy controls without confounding ophthalmic conditions we also enrolled. For the NAION prediction task, the dataset included 327 eyes: 180 contralateral eyes from unilateral NAION patients and 147 eyes from normal controls. For the task to discriminate NAION from acute ON, the dataset included 180 eyes: 87 acute NAION eyes and 93 acute ON eyes.

All eyes underwent OCTA examinations with paired clinical parameters relevant to NAION pathogenesis.

To address the within-subject correlation introduced by including paired eyes from the same NAION patients, we employed patient-level stratification during all cross-validation splits: all eyes from a single patient were assigned to the same cross-validation fold, ensuring that the training and testing data were fully independent at the patient level, thus eliminating information leakage and accounting for correlated observations within the same subject. Additionally, we utilized mixed-effects models in our preliminary statistical analyses to adjust for the clustering of eyes within patients.

### Optical coherence tomography angiography(OCTA)

**Fig. 10:**
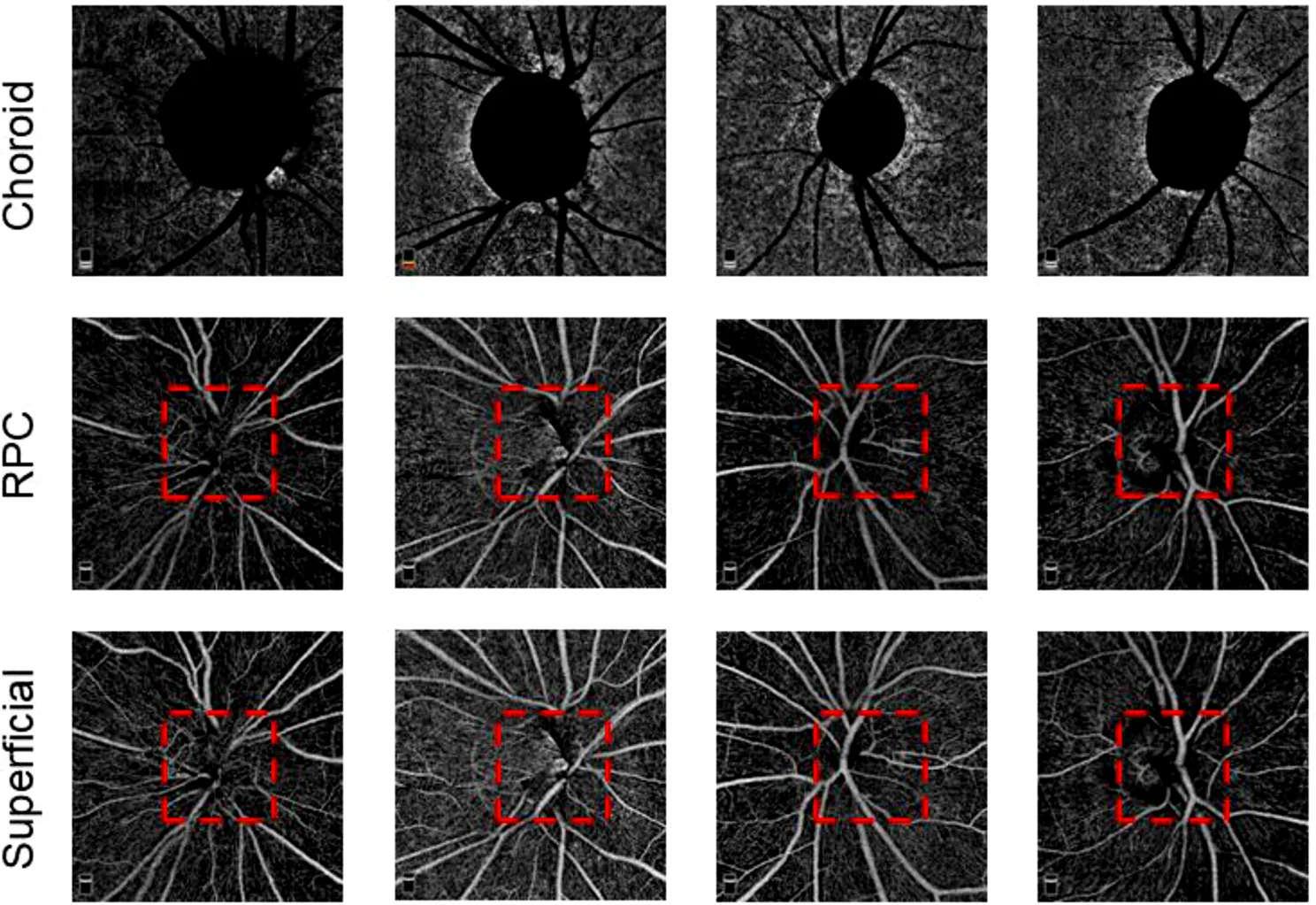
En face projections of three peripapillary OCTA vascular plexuses. A: Acute NAION; O: Acute optic neuritis; H: Healthy eye; N: Normal control eye

OCTA acquisitions were performed using the RTVue-XR Avanti OCTA system (software version 2.0.5.39). This device noninvasively captures high-resolution microvascular information across multiple retinal layers, including the choroidal layer, radial RPC layer, and superficial retinal plexus. (Fig. 10) Each subject’s OCTA volume included perfusion data from these three critical vascular plexuses, providing multiple perspectives for both NAION prediction and acute-phase differentiation between NAION and ON.

Table 2 demonstrates four ocular conditions in the two classification tasks: acute NAION, acute ON, contralateral eyes of unilateral NAION patients (designated ‘Healthy’), and normal control eyes (‘Normal’). All the images were standardized to 400 × 400 pixels with 8-bit grayscale (0–255) representing perfusion density and vascular morphology.

### Clinical information (C-Info)

To systematically identify clinically relevant factors associated with NAION pathogenesis, we documented nine key vascular risk parameters beyond age and sex, namely, diabetes mellitus, hypertension, dyslipidaemia, obstructive sleep apnoea (OSA), smoking status, and alcohol consumption. Consistent with established evidence [17, 18], NAION patients exhibit both anatomic disc risk factors (e.g., small cup-to-disc ratio) and systemic vascular comorbidities such as hypertension, diabetes, dyslipidaemia, obesity, tobacco use, and OSA.

These parameters were incorporated into the model development and internal validation for classification efficacy. Intergroup comparisons confirmed the following: no significant difference in age (ANOVA, *P* = 0.577) and no significant difference in sex distribution (χ² test, *P* = 0.126).

## Results

### Diagnostic model performance evaluation

Table 6 presents the results for NAION prediction and acute-phase differentiation between NAION and ON. The sudden vision loss in NAION makes it difficult to capture preonset ocular states, highlighting the importance of early prediction for timely intervention to reduce disease severity. We used the contralateral unaffected eyes of unilateral NAION patients to simulate preonset states and established an MLP-based NAION prediction model with OCTA data from these eyes versus healthy controls.

**Table 6:** Diagnostic performance of multimodal OCTA radiomics models for NAION identification and differentiation

Task	Methods	AUC (95% CI)	ACC (95% CI)	F1 (95% CI)	PREC (95% CI)	REC (95% CI)	SPE (95% CI)
NAION Identification	RPC	0.831 (0.787, 0.875)	0.758 (0.709, 0.801)	0.778 (0.732, 0.824)	0.840 (0.774, 0.889)	0.724 (0.655, 0.784)	0.805 (0.734, 0.861)
	Superficial	0.843 (0.801, 0.885)	0.778 (0.730, 0.820)	0.796 (0.752, 0.840)	0.860 (0.798, 0.906)	0.741 (0.673, 0.799)	0.829 (0.760, 0.882)
	Superficial + C-Info	0.943 (0.918, 0.968)	0.859 (0.818, 0.893)	0.868 (0.829, 0.907)	0.958 (0.909, 0.981)	0.793 (0.729, 0.845)	0.951 (0.899, 0.977)
	RS + C-Info	0.996 (0.983, 1.000)	0.970 (0.945, 0.984)	0.974 (0.951, 0.987)	0.982 (0.946, 0.994)	0.966 (0.925, 0.985)	0.976 (0.936, 0.991)
NAION with Acute ON	Choroid	0.890 (0.840, 0.940)	0.778 (0.711, 0.833)	0.760 (0.692, 0.828)	0.760 (0.666, 0.835)	0.760 (0.666, 0.835)	0.793 (0.699, 0.864)
	RPC	0.822 (0.760, 0.884)	0.759 (0.691, 0.816)	0.735 (0.664, 0.806)	0.750 (0.651, 0.829)	0.720 (0.621, 0.801)	0.793 (0.699, 0.864)
	Superficial	0.810 (0.746, 0.874)	0.722 (0.652, 0.783)	0.694 (0.619, 0.769)	0.708 (0.606, 0.792)	0.680 (0.579, 0.765)	0.759 (0.662, 0.835)
	Choroid + C-Info	0.935 (0.897, 0.973)	0.870 (0.814, 0.912)	0.863 (0.805, 0.921)	0.846 (0.760, 0.906)	0.880 (0.799, 0.931)	0.862 (0.778, 0.918)
	CRS + C-Info	0.989 (0.966, 1.000)	0.944 (0.896, 0.971)	0.941 (0.893, 0.971)	0.923 (0.848, 0.963)	0.960 (0.896, 0.985)	0.931 (0.859, 0.968)

First, radiomics features extracted from OCTA layers were analysed. Features with absolute correlation coefficients > 0.3 were retained for modelling. No discriminative radiomics features were found in the choroidal layer; thus, the analysis focused on the radial RPC and retinal layers. The single-modality single-region model using retinal-layer OCTA features outperformed the RPC model.

The addition of clinical information (C-Info) related to NAION onset to retinal radiomics features further improved the performance of the multimodal single-region NAION prediction model. To support clinical decision-making, we integrated radiomics features from multiple OCTA regions and clinical information to construct a multimodal multiregional model to achieve optimal NAION prediction.

In the discrimination task between acute-phase NAION and acute-phase ON under identical correlation thresholds and training strategies, radiomics features were identified across all three OCTA layers: the choroid, RPC, and superficial retina. The choroidal single-modality single-region model performed best—in contrast to NAION prediction. The multimodal choroidal model (with C-Info) further enhanced differentiation performance. The multimodal multiregion model combining all the features achieved peak effectiveness.

Decision patterns in both tasks aligned with clinical diagnoses, demonstrating the necessity of multiperspective information integration for disease diagnosis.

**Fig. 11:**
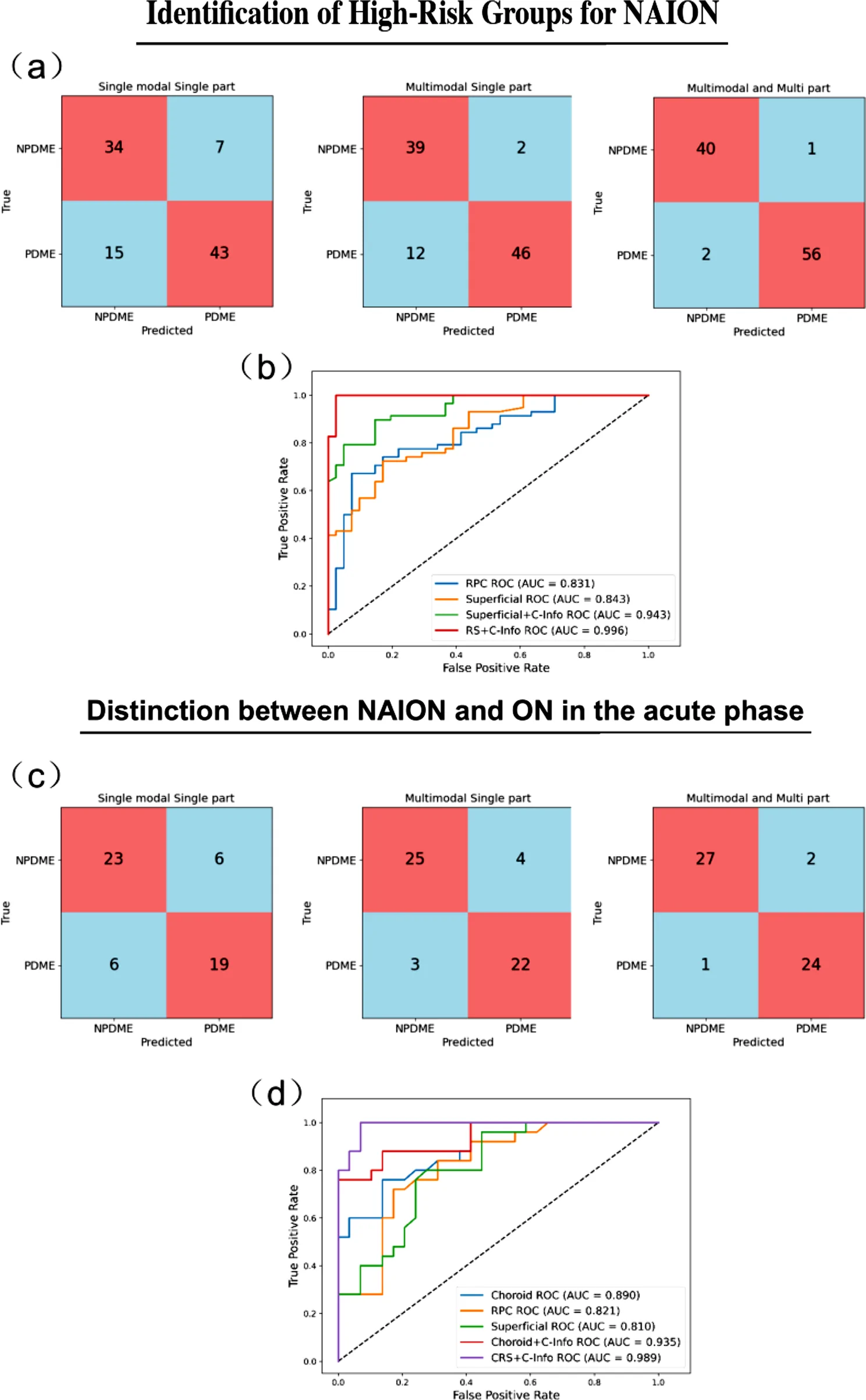
Confusion matrices and ROC curves for two NAION diagnostic tasks. Panels (**a**) and (**b**) present the confusion matrix and ROC curve for the NAION high-risk population identification task, respectively. Panels (**c**) and (**d**) present the confusion matrix and ROC curve for acute NAION vs. acute optic neuritis (ON) differentiation, respectively. Results are shown for three modelling strategies: single-modality single-region, multimodal single-region, and multimodal multiregion, integrating OCTA radiomics features and clinical information

Figure 11 displays the confusion matrices and ROC curves for both tasks using models built with OCTA radiomics features alone and combined with clinical information, the results of which are consistent with the above conclusions.

Figure 11(a) and (b) show the confusion matrix and ROC curve, respectively, for the NAION identification task. The best single-modality single-region model achieved an AUC of 0.843 and an ACC of 0.778. The multimodal single-region model incorporating clinical information with retinal-layer radiomics features achieved an AUC of 0.943 and an ACC of 0.859. The multimodal multiregion prediction model combining radiomics features from the RPC and retinal layers with clinical information achieved the highest AUC of 0.996 (95% CI: 0.983–1.000) and ACC of 0.970, indicating excellent performance. Consistent with the three modelling approaches in the identification of high-risk NAION populations task, Fig. 11(c) and (d) show the confusion matrix and ROC curve, respectively, for the discrimination task between acute-phase NAION and acute-phase ON. The AUC and ACC values for the different models were 0.890 and 0.778, 0.935 and 0.870, and 0.989 (95% CI: 0.966–1.00) and 0.944, respectively.

The confusion matrices reveal that the diagnostic accuracy for all categories improves as more OCTA regions and multimodal information are integrated.

### Performance comparison of the MLP multimodal fusion model with the other models

**Fig. 12:**
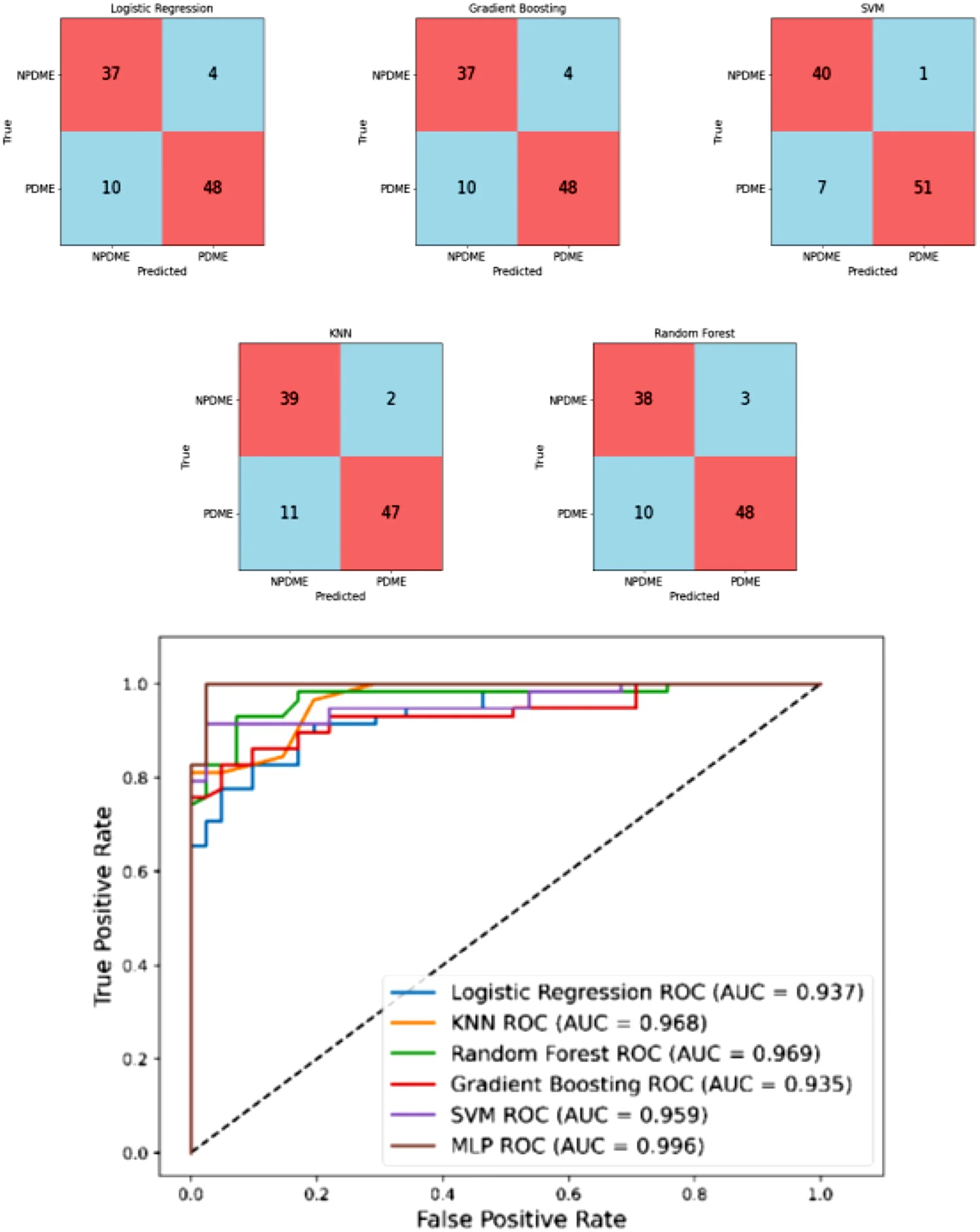
Identification of high-risk NAION populations. Left panel: Confusion matrices of the different models. Right panel: ROC curves of the different models

**Fig. 13:**
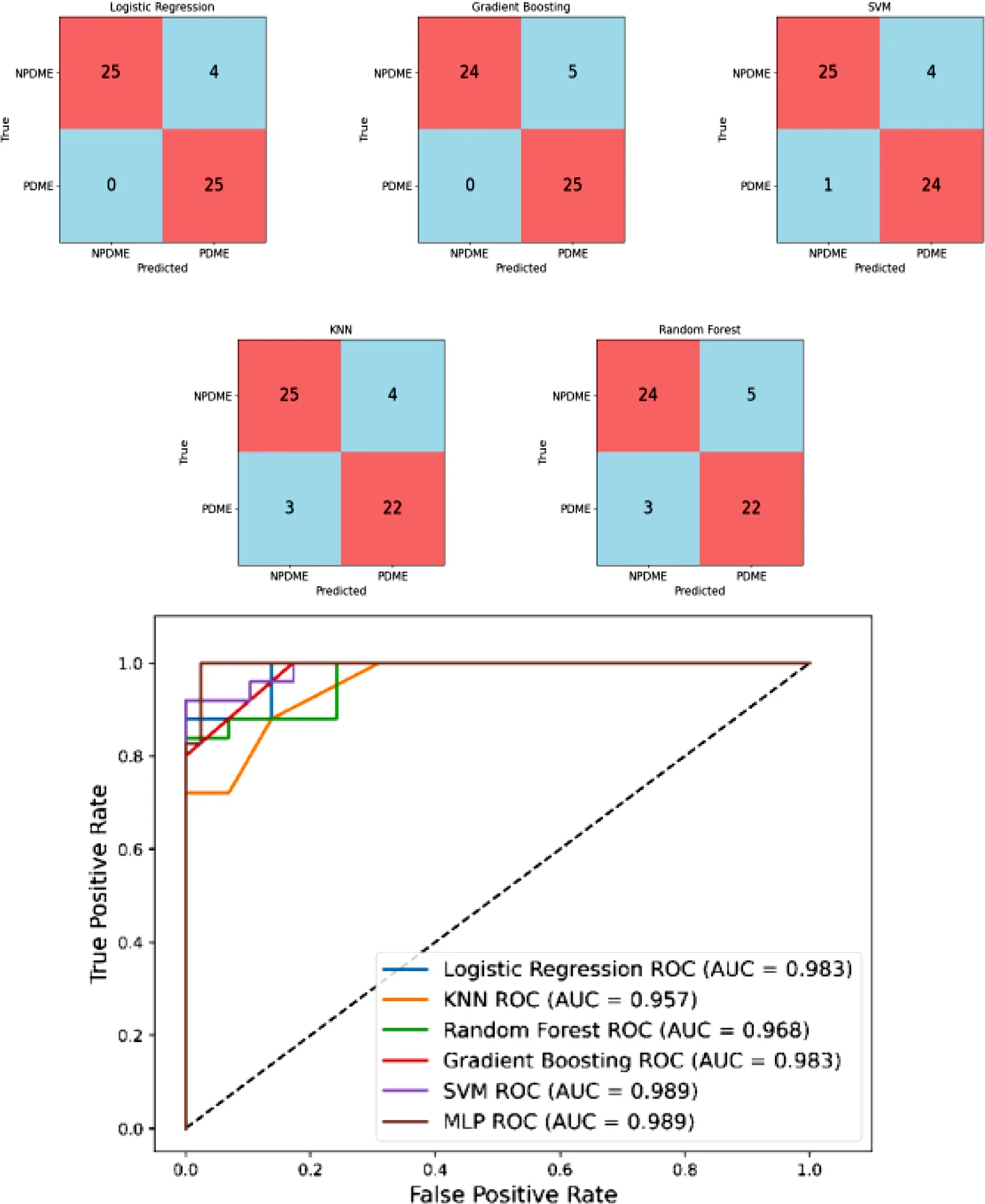
Discrimination task between acute-phase NAION and acute-phase ON. Left panel: Confusion matrices of the different models. Right panel: ROC curves of the different models

In addition to investigating the MLP model, this study also validated other models, including logistic regression, K-nearest neighbour (KNN), random forest, gradient boosting, and SVM. In the performance comparison across models, the MLP multimodal fusion model demonstrated superior overall performance in both tasks. Table 7; Fig. 12 show the identification results for high-risk NAION populations, whereas Table 8; Fig. 13 show the results of the differentiation task between acute-phase NAION and acute-phase ON. The model outperformed all the other models in terms of the ACC, F1 score, and balance.

**Table 7:** Diagnostic performance of the different models in the identification of high-risk NAION populations

Model	Methods	AUC (95% CI)	ACC (95% CI)	F1 (95% CI)	PREC (95% CI)	REC (95% CI)	SPE (95% CI)
L R	RS + C-Info	0.937 (0.910, 0.964)	0.856 (0.814, 0.890)	0.873 (0.832, 0.914)	0.923 (0.871, 0.955)	0.828 (0.766, 0.876)	0.902 (0.843, 0.940)
KNN	RS + C-Info	0.968 (0.950, 0.986)	0.869 (0.828, 0.902)	0.879 (0.839, 0.919)	0.959 (0.915, 0.981)	0.810 (0.747, 0.860)	0.951 (0.901, 0.976)
R F	RS + C-Info	0.969 (0.951, 0.987)	0.869 (0.828, 0.902)	0.881 (0.841, 0.921)	0.941 (0.893, 0.969)	0.828 (0.766, 0.876)	0.927 (0.873, 0.959)
G B	RS + C-Info	0.935 (0.908, 0.962)	0.858 (0.816, 0.892)	0.873 (0.832, 0.914)	0.923 (0.871, 0.955)	0.828 (0.766, 0.876)	0.902 (0.843, 0.940)
S V M	RS + C-Info	0.959 (0.938, 0.980)	0.919 (0.884, 0.945)	0.876 (0.832, 0.920)	0.978 (0.938, 0.992)	0.793 (0.729, 0.845)	0.976 (0.936, 0.991)
MLP	RS + C-Info	0.996 (0.983, 1.000)	0.970 (0.945, 0.984)	0.974 (0.951, 0.987)	0.982 (0.946, 0.994)	0.966 (0.925, 0.985)	0.976 (0.936, 0.991)

RS represents RPC+superficial, and C-Info represents clinical information

**Table 8:** Diagnostic performance of different models in the discrimination task between acute-phase NAION and acute-phase ON

Model	Methods	AUC (95% CI)	ACC (95% CI)	F1 (95% CI)	PREC (95% CI)	REC (95% CI)	SPE (95% CI)
L R	CRS + C-Info	0.983 (0.964, 1.000)	0.925 (0.877, 0.956)	0.926 (0.877, 0.961)	0.862 (0.778, 0.918)	1.000 (0.958, 1.000)	0.862 (0.778, 0.918)
KNN	CRS + C-Info	0.957 (0.924, 0.990)	0.870 (0.814, 0.912)	0.863 (0.805, 0.921)	0.846 (0.760, 0.906)	0.880 (0.799, 0.931)	0.862 (0.778, 0.918)
R F	CRS + C-Info	0.968 (0.940, 0.996)	0.851 (0.793, 0.896)	0.846 (0.785, 0.907)	0.815 (0.725, 0.879)	0.880 (0.799, 0.931)	0.828 (0.740, 0.890)
G B	CRS + C-Info	0.983 (0.964, 1.000)	0.907 (0.856, 0.942)	0.909 (0.858, 0.948)	0.833 (0.745, 0.895)	1.000 (0.958, 1.000)	0.828 (0.740, 0.890)
S V M	CRS + C-Info	0.989 (0.973, 1.000)	0.907 (0.856, 0.942)	0.905 (0.853, 0.945)	0.857 (0.773, 0.914)	0.960 (0.896, 0.985)	0.862 (0.778, 0.918)
MLP	CRS + C-Info	0.989 (0.966, 1.000)	0.944 (0.896, 0.971)	0.941 (0.893, 0.971)	0.923 (0.848, 0.963)	0.960 (0.896, 0.985)	0.931 (0.859, 0.968)

CRS represents Choroid + RPC+superficial, C-Info represents clinical information

### Clinical physicians vs. AI models: controlled experiment

To compare the performance of the model with that of clinical experts, two neuro-ophthalmologists with more than 10 years (Physician 1) and 25 years (Physician 2) of experience in optic nerve disorders were included. The experts were provided with exactly the same input information as the model: the full set of OCTA images (including superficial, RPC, and choroidal layers) and the patients’ basic clinical information (age, sex, and relevant medical history). All the experts were fully blinded to the final clinical diagnoses of the patients, and they evaluated the independent test set samples that were not used in model training, ensuring a fair and unbiased comparison.

The two experts independently classified eyes in the identification of high-risk NAION populations model (healthy contralateral eyes vs. normal controls) and the acute-phase discrimination model (acute NAION vs. acute ON). F1 scores and other metrics derived from these classifications were analysed and compared against the performance of the AI models. The Results for the identification of high-risk NAION populations are shown in Table 9 and are as follows: Physician 1: F1 score 0.677 (95% CI, 0.629–0.725) and Physician 2: F1 score 0.750 (95% CI, 0.704–0.796). For the discrimination task between NAION and acute-phase ON, the results are shown in Table 10 and are as follows: Physician 1: F1 score 0.782 (95% CI, 0.779–0.785) and Physician 2: F1 score 0.861 (95% CI, 0.858–0.864).

**Table 9:** Clinician performance in the identification of high-risk NAION populations

	ACC (95% CI)	F1 (95% CI)	PREC (95% CI)	REC (95% CI)	SPE (95% CI)
Physician 1	0.611 (0.557, 0.662)	0.677 (0.629, 0.725)	0.579 (0.517, 0.638)	0.815 (0.752, 0.865)	0.407 (0.331, 0.488)
Physician 2	0.741 (0.691, 0.785)	0.750 (0.704, 0.796)	0.724 (0.657, 0.782)	0.778 (0.712, 0.833)	0.704 (0.626, 0.772)

**Table 10:** Clinician performance in discriminating between NAION and acute-phase ON

	ACC (95% CI)	F1 (95% CI)	PREC (95% CI)	REC (95% CI)	SPE (95% CI)
Physician 1	0.722 (0.652, 0.782)	0.782 (0.779, 0.785)	0.900 (0.805, 0.951)	0.692 (0.589, 0.779)	0.706 (0.607, 0.789)
Physician 2	0.792 (0.727, 0.845)	0.861 (0.858, 0.864)	0.850 (0.761, 0.910)	0.872 (0.786, 0.927)	0.571 (0.470, 0.667)

In this study, the MLP fusion model outperformed clinicians with 10 and 25 years of experience across all the metrics. This AI model maintained exceptionally high accuracy with large-scale data, demonstrating robust quantitative analysis capabilities, particularly in detecting subtle distinctions imperceptible to the human eye. In contrast, clinicians faced diagnostic errors under high-volume data conditions because of highly overlapping clinical phenotypes in the images. The integration of radiomics and deep learning now provides critical clinical decision-making support, including early warning systems, disease differentiation and dynamic tracking of disease progression.

## Discussion

According to Zhong et al. [19], compared with normal control eyes, the fellow unaffected eyes of NAION patients exhibit reduced peripapillary capillary density, with a particularly significant decrease in the superficial peripapillary capillary plexus density. Since the fellow eyes share identical risk factors and similar optic disc characteristics, they face an elevated risk of developing NAION. This phenomenon provides critical insights into the pathological mechanisms of NAION, suggesting that the disease process involves not only the affected eye but may also reflect underlying microcirculatory abnormalities or haemodynamic alterations already present in the clinically unaffected contralateral eye. Furthermore, research by Collignon-Robe et al. [20] confirmed distinct microvascular differences between NAION and ON. Their study quantified circulatory abnormalities in the optic nerve head between these conditions and evaluated the potential of these measurements to differentiate acute NAION from acute ON. The findings demonstrated that the reduction in erythrocyte flow velocity width within the optic nerve head capillaries was significantly greater in NAION patients than in ON patients. This comparative analysis established a potential basis for the use of imaging modalities to distinguish between acute NAION and acute ON in future clinical practice.

The diagnostic workup for NAION integrates both conventional and advanced modalities. The core imaging triad includes fundus photography (baseline documentation), fluorescein angiography (FFA) (dynamic vascular assessment), and quantitative perimetry (visual field/VF analysis). OCT structural biomarkers include pRNFL thickness and ganglion cell complex (GCC) metrics in the macular region [3]. Over the past decade, OCTA has undergone progressive refinement. Utilizing motion contrast principles, this noninvasive modality enables high-resolution quantification of vessel density (VD) in both the optic nerve head and macula. In acute NAION, OCTA reveals segmental or diffuse VD reduction within the peripapillary capillaries and macular deep vascular plexus. This hypoperfusion progressively attenuates in the chronic phase, representing a transformative advancement in NAION diagnostics.

Quantifying peripapillary microvascular alterations in optic neuropathies remains challenging. Fundus fluorescein angiography (FFA) is invasive and cannot resolve superimposed vascular plexuses. Although OCTA enables noninvasive vascular mapping, its accuracy is compromised during the acute phases of NAION and ON owing to motion artefacts and oedema-induced signal attenuation [21]. CAD refers to an integrated system that leverages medical imaging informatics, biomarker quantification, and computational algorithms to detect pathological features and enhance diagnostic precision. Its core function resides in transforming subjective interpretation into objective decision support through three-tiered analysis [22]. Recent advances in AI have accelerated this progress, with CAD models now achieving diagnostic accuracy comparable to, if not superior to, that of subspecialty clinicians [23]. This identical computational framework has been adapted for diagnostics in ophthalmic pathologies [17, 18, 24]. The superior visualization of fundal vasculature by OCTA, coupled with the efficacy of CAD in extracting and analysing critical features from medical images, enables both efficient prediction of NAION and highly accurate differentiation between NAION and ON during the acute phase. Zhong et al. [19] demonstrated that OCTA reveals significantly reduced perfusion density in the RPC and peripapillary superficial capillary plexus among NAION-affected eyes, contralateral unaffected eyes, and normal controls. Bunod et al. [6] established a multimodal OCTA model that integrates data from both the RPC and superficial capillary plexus regions, achieving significantly better diagnostic performance than single-region models. While demonstrating exceptional efficacy in discriminating patients with NAION, patients with glaucoma, and normal controls, their contribution focused primarily on differentiating among these three entities.

In traditional neuro-ophthalmic diagnosis, the primary approach combines imaging examinations such as OCTA, visual field testing, GCC, RNFL, and fundus photography with clinical information and patient signs. However, various conventional examinations currently face limitations: OCTA vessel density measurements vary across different algorithms and demonstrate insensitivity to early-stage biomarkers. Visual field testing is heavily dependent on subjective factors requiring reliable patient cooperation, but optic nerve disease patients often have suboptimal compliance. GCC analysis is constrained by a temporal window of detectability; its sensitivity is insufficient during early disease stages, requiring 3 to 6 weeks post-onset to show significant changes [25–27]. In cases of acute disc oedema, RNFL imaging suffers from limited layer resolution, making it impossible to differentiate between true nerve fibre loss and axonal swelling, thereby compromising prognostic evaluation [25].

Radiomics and convolutional neural networks (CNNs) [28] in deep learning, along with vision transformers (ViT) [29], represent two prevalent approaches for image feature extraction. ImageNet [30], a large-scale visual recognition challenge encompassing tasks such as image classification, has spawned high-performance models, including GoogLeNet [31], ResNet [18] and VGG [32]. While these CNN-based architectures have progressively surpassed human-level performance in image classification through iterative refinements, their intrinsic limitations impede model development for NAION prediction and acute-phase differentiation between NAION and ON, as outlined below:.

(i) Training deep neural networks for disease discrimination based on imaging examinations typically demands substantial datasets—often unattainable in clinical practice, particularly for conditions such as NAION. Limited data availability, compounded by complex network structures and excessive parameters, hinders model optimization and compromises robustness;.
(ii) OCTA scans from NAION patients exhibit minimal discernible differences even for clinical experts, in contrast to natural images from ImageNet, where interclass features are distinctly evident. Although ViT networks [29] adapted from the transformer architecture excel at capturing global image features compared with CNNs, they are ill suited for extracting fine-grained features in OCTA imagery. Consequently, we employed radiomics for OCTA feature extraction.

In contrast, radiomics features typically possess explicit mathematical definitions and physical interpretations—such as shape and texture features—ensuring interpretability [33].

The sudden vision loss associated with NAION makes it difficult to take preventive measures in advance and obtain the microvascular status of the eye immediately before NAION onset to establish predictive models for at-risk patients. However, the microvascular status of the contralateral unaffected eye of unilateral NAION patients differs from that of normal eyes. These changes approximate the microvascular state immediately preceding NAION onset and indicate increased susceptibility. Therefore, we utilize the contralateral unaffected eyes of unilateral NAION patients to simulate the preonset ocular state to construct a model for identifying high-risk NAION populations. This enables screening and prediction for potential NAION patients who have not yet developed symptoms, thereby significantly reducing disease severity and facilitating early intervention.

NAION and ON are distinct optic neuropathies. Their ocular manifestations during the acute phase are highly similar, making clinical differentiation by experts challenging. This increases misdiagnosis rates and may lead to inappropriate therapeutic regimens.

While clinicians routinely use valuable physical examination findings (pain with eye movement, or RAPD) to differentiate NAION from ON, our AI pipeline offers the following complementary advantages:

Objective Quantification: Our multimodal OCTA radiomics model is designed to serve as a clinical decision-support tool rather than a replacement for expert clinical judgement. We envision three primary clinical scenarios where this model could add value:

Scenario 1: Community screening and primary care settings: In settings without access to neuro-ophthalmology specialists, the model could be used to analyse OCTA images obtained during routine eye examinations. Patients identified as high risk for NAION could be prioritized for referral and closer monitoring, facilitating earlier intervention.

Scenario 2: Emergency department and acute care settings: For patients presenting with acute vision loss and disc oedema, the model could assist in the rapid differentiation between acute NAION and acute ON. This could help guide initial management decisions and reduce unnecessary invasive procedures (e.g., excessive MRI scanning and lumbar puncture) when NAION is strongly suspected.

Scenario 3: Follow-up and monitoring: In patients with known NAION risk factors or a history of unilateral NAION, the model could be used for longitudinal monitoring of the contralateral eye to detect subtle microvascular changes that may precede clinical onset.

In all scenarios, the model output should be interpreted as an adjunct and not a substitute for comprehensive clinical evaluation, including medical history, physical examination, and ancillary testing. The final diagnosis and treatment decisions should always be made by qualified ophthalmologists.

Unlike subjective clinical assessment, the radiomics-based approach yields quantitative, standardized, and reproducible measurements that minimize interreader variability. Furthermore, the model can identify microvascular alterations imperceptible to human readers, potentially enabling earlier detection of at-risk individuals. Automated analysis of OCTA images can be performed efficiently, a feature that is particularly valuable in high-volume clinical settings. Finally, compared with invasive techniques such as fluorescein angiography, OCTA, which is a noninvasive imaging modality, renders serial monitoring and population-level screening more feasible.

On OCTA, the choroid, peripapillary capillary layer, and retinal layers provide distinct perspectives for observing ocular conditions. Using radiomics, we extracted features from these individual regions and selected highly relevant features for model development. Combined with NAION-related clinical information, we constructed three MLP-based models—single-modality single-region, multimodality single-region, and multimodality multiregion—to investigate how OCTA examinations of these three regions impact two tasks: identification of high-risk NAION populations and discrimination between acute-phase NAION and acute-phase ON.

Our integration of multiregion OCTA data and clinical information successfully addressed both tasks, demonstrating alignment with the diagnostic decision-making process of clinical experts. Consequently, the high accuracy achieved in both the NAION prediction task and the NAION/ON acute-phase differentiation task enables timely administration of appropriate treatments, reduces NAION severity, and improves postinfarct prognosis.

Limitations: First, the very high AUC values, particularly for the identification of high-risk NAION populations, should be interpreted with caution despite our internal cross-validation strategy and supportive shuffle test. Specifically, we acknowledge that the exceptionally high AUC (0.996) may carry a risk of overfitting to the idiosyncrasies of this single-centre cohort and potential spectrum bias due to the age- and sex-matched study population, which may not fully reflect the broader clinical population with diverse comorbidities and disease presentations. These results are promising but indicative and may represent an upper bound of model performance achievable within this specific cohort.

Second, we note that OCTA imaging in the context of acute disc oedema has inherent limitations that may affect feature extraction: acute tissue swelling can lead to signal attenuation in deeper vascular layers, which may reduce the quality of choroidal layer imaging; and motion artefacts, which may occur due to impaired fixation in patients with acute visual loss and can introduce noise into the images, thereby introducing variability in the extracted radiomics features. While we excluded low-quality images from our analysis to mitigate these issues, these factors remain potential sources of variability that need to be addressed in future larger-scale studies with more robust image preprocessing techniques.

The limited and likely single-centre sample size increases the risk of learning centre-specific biases. The reported performance might not fully generalize to other populations with different imaging protocols, patient demographics, or disease prevalences.

The most significant limitation of this study is the absence of an external validation cohort. Future work must prioritize the recruitment of patients from multiple independent clinical centres to validate the performance and robustness of the model.

## Conclusions

This study presents an OCTA-based multimodal fusion model for the assessment of NAION. By integrating radiomics features extracted from en face images of the retinal superficial layer, choroidal layer, and radial peripapillary capillary plexus in the optic disc region, combined with systemic clinical risk factors, we developed a diagnostic model based on a MLP architecture. The model addresses two key unmet clinical needs: the identification of high-risk NAION populations and the discrimination between acute-phase NAION and acute-phase ON. In the present single-centre cohort, the diagnostic performance of the model was favourable and exceeded that of experienced neuro-ophthalmologists; thus, the model represents a promising AI-assisted decision-making support tool for neuro-ophthalmologists.

The potential clinical value and implications of this model are summarized as follows:

(i) Diagnostic scope: The model enables risk estimation of NAION in asymptomatic patients and supports differential diagnosis between acute-phase NAION and acute-phase ON accompanied by optic disc oedema.
(ii) Diagnostic accuracy: By fusing multisource imaging and clinical information, the model achieved high diagnostic performance in the tested cohort, with the potential to complement and enhance conventional diagnostic workflows.
(iii) Clinical decision-making support: The standard diagnostic workup for NAION typically requires a panel of examinations, including OCTA, fundus fluorescein angiography, pRNFL measurement, visual field testing, fundus photography, and, in selected cases, neuroimaging and intracranial pressure assessment. With only OCTA images and basic clinical data as inputs, this model has the potential to reduce redundant examinations, shorten diagnostic turnaround time, and optimize clinical resource allocation, providing an additional decision-making perspective for clinicians.
(iv) Early intervention support: Accurate early risk stratification and timely differential diagnosis may facilitate earlier intervention and personalized management for patients with or at risk of NAION, potentially mitigating disease progression and improving long-term visual prognosis.
(v) Population-level screening: The model provides an objective, noninvasive approach for NAION risk screening, which may improve screening efficiency in primary care and community-based settings.
(vi) Methodological innovation: This study establishes a radiomics-driven multimodal diagnostic framework, offering a novel methodological pathway for the early detection and management of NAION and other optic neuropathies.

Importantly, the clinical use of this model requires further rigorous validation. The current model was developed and internally validated in a single-centre retrospective cohort, and its generalizability across diverse ethnic populations, imaging device platforms, scanning protocols, and clinical centres remains to be verified. Large-scale, multicentre external validation and prospective real-world clinical trials are needed before this model can be integrated into routine clinical practice.

Future work will prioritize multicentre external validation, algorithm optimization for heterogeneous imaging data, and prospective real-world performance evaluation to steadily promote the clinical translation of this diagnostic model.

## Data Availability

This retrospective study utilized de-identified medical images stripped of all personally identifiable information.All data were obtained from the Department of Ophthalmology, The First Affiliated Hospital of Kunming Medical University.The raw data supporting the conclusions of this article will be made available by the authors, without undue reservation.
